# Evaluation and Selection of Rice Cultivars for Dual-Purpose Grain and Forage Production System

**DOI:** 10.3390/plants15172725

**Published:** 2026-09-06

**Authors:** Guangyi Chen, Lin Yang, Li Zhang, Conghua Zhu, Wei Li, Hong Yang, Ziyu Li, Yao Zhang, Banglin Xiao, Junqi Yu, Tian Li, Xuyi Li, Yuyuan Ouyang

**Affiliations:** 1 Crop Research Institute of the Sichuan Academy of Agricultural Sciences, Chengdu 610066, China; chenguangyi@scsaas.cn (G.C.);; 2 Sichuan Provincial Key Laboratory of Green Germplasm Innovation and Genetic Improvement for Grain and Oil Crops, Chengdu 611130, China; 3 College of Agronomy, Chengdu Agricultural College, Chengdu 611130, China; 4 College of Agronomy, Sichuan Agricultural University, Chengdu 611130, China

**Keywords:** silage rice, ratoon rice, yield, quality, dual-purpose grain and forage production system

## Abstract

Ratoon rice is the production of a second rice crop from the stubble left behind after the main crop has been harvested. To identify suitable ratoon rice cultivars for a dual-purpose grain and forage production system, 25 widely cultivated rice cultivars in southwestern China were systematically evaluated based on ratooning ability, forage productivity, ratoon rice yield, and Cd accumulation characteristics. Significant variations were observed among the tested cultivars, indicating substantial differences in their suitability for this production system. Principal component analysis identified three cultivars, CKY637, CKYSM, and FY498, as promising candidates because of their favorable combination of ratooning ability (1.33–1.61), silage rice yield (8.85–11.27 t ha^−1^), ratoon rice yield (7.83–9.65 t ha^−1^), and low grain Cd accumulation (0.0430–0.0457 mg kg^−1^). Among them, CKY637 achieved the highest comprehensive evaluation score, whereas CKYSM exhibited particularly stable performance in maintaining low Cd accumulation, and FY498 was well balanced between yield and quality. These results suggest that cultivar suitability for the dual-purpose system depends on the coordinated performance of multiple traits, particularly strong ratooning ability, high forage and ratoon rice productivity, short total growth duration, and low Cd accumulation in ratoon rice grains. Overall, CKY637, CKYSM, and FY498 represent candidate cultivar resources for the first-season forage + second-season ratoon rice system and provide practical materials for its application in subtropical rice-growing regions.

## 1. Introduction

**Figure 1:**
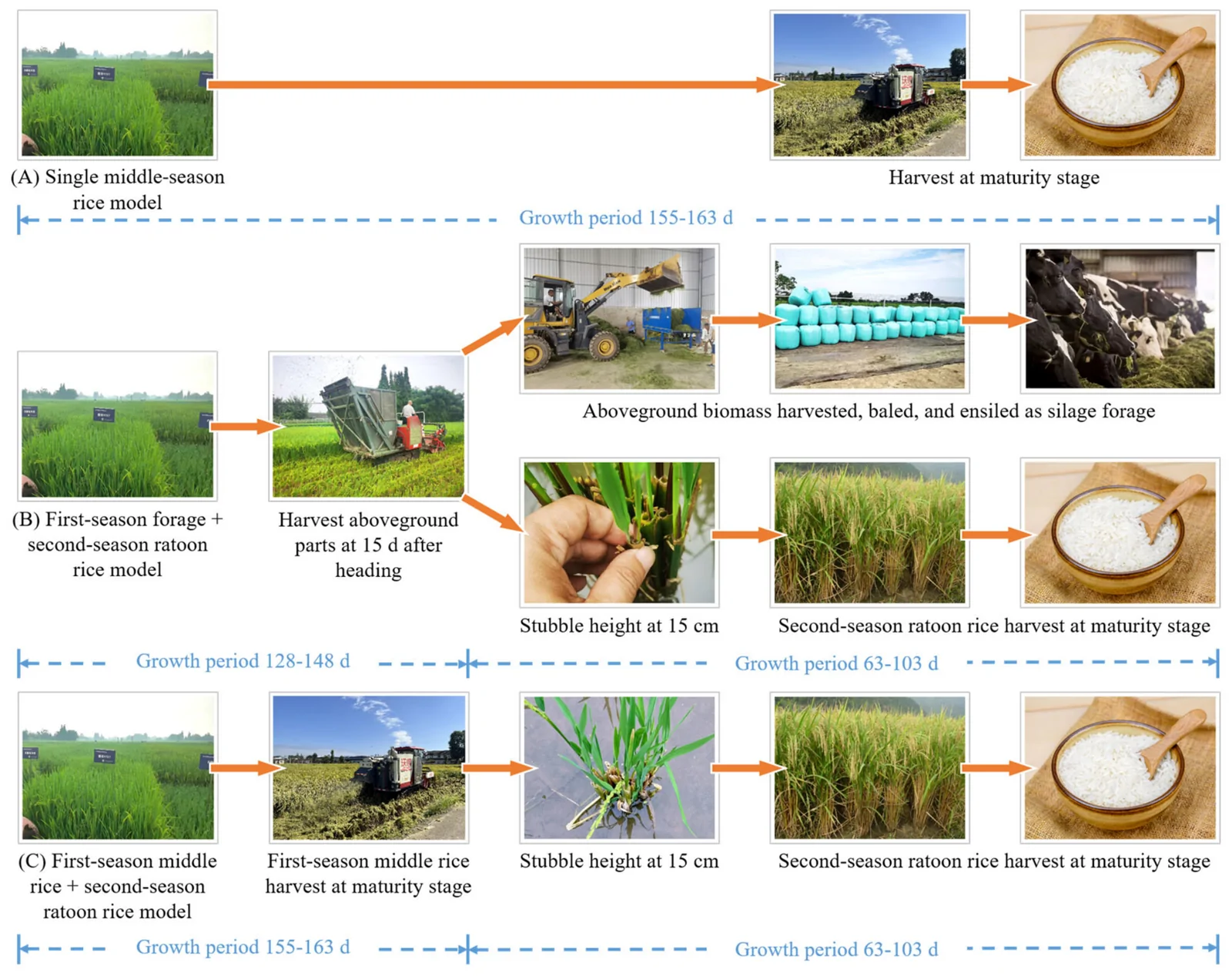
Representative photographs showing the key growth stages of rice under different cultivation systems.

Ratoon rice is an efficient multiple-cropping system that utilizes the regenerative ability of axillary buds on rice stubble after the first-season harvest to produce a second crop (Figure 1C) [1]. In recent decades, climate warming has substantially improved the thermal conditions for ratoon rice production in the Sichuan Basin. Previous studies have shown that, from 1961 to 2020, the safe growing season, accumulated active temperature, and safe grain-filling period for ratoon rice all increased significantly, particularly in the western Sichuan Plain. Consequently, the suitable cultivation area for ratoon rice has expanded northward and westward, with approximately 20% of the Sichuan Basin being reclassified as moderately suitable for ratoon rice cultivation during 1991–2020 [2]. By producing both a first-season and a ratoon crop without increasing cultivated land, the ratoon rice system effectively enhances the cropping index, contributing to stable grain production, higher farmer income, reduced carbon emissions, and sustainable agricultural development, thereby representing an important strategy for ensuring food security [3]. Furthermore, previous studies have suggested that considerable yield potential remains untapped, with theoretical yield increases of 3.38, 3.27, and 5.41 t ha^−1^ for the first-season crop, ratoon crop, and total annual production, respectively [4].

However, heavy metal contamination, particularly cadmium (Cd), has become one of the major constraints to sustainable rice production in China. In populations where rice is the staple food, rice grain represents one of the primary dietary sources of Cd exposure [5,6]. Soil Cd contamination not only reduces crop productivity but also increases the grain Cd concentration, posing serious risks to food safety and human health [7]. Various remediation strategies have been developed to mitigate Cd contamination in paddy fields, including chemical remediation [8], phytoremediation [9], agronomic management [10], and cultivar selection [11]. Among these approaches, the selection and breeding of low-Cd-accumulating rice cultivars is considered one of the most practical, economical, and environmentally friendly strategies for reducing grain Cd accumulation [12]. Previous studies have demonstrated that Cd exhibits distinct tissue-specific distribution patterns in rice plants, with its accumulation differing between first-season and ratoon crops. Compared with the first-season crop, the ratoon crop generally exhibits substantially lower grain Cd concentrations because a greater proportion of Cd is retained in the roots, stubble, and vegetative tissues [13,14]. Similar phenomena have also been reported in ratooned tobacco and chicory [15,16,17], suggesting that ratoon growth may alter the uptake, translocation, and partitioning of heavy metals and mineral elements within plants [18,19].

Despite its considerable production potential, ratoon rice cultivation in Sichuan Province still faces several challenges. Increasing Cd contamination threatens grain safety, while the supply of high-quality rice from the single middle-season crop remains insufficient to satisfy the growing consumer demand for premium rice. At the same time, the rapid expansion of the livestock industry has intensified the shortage of high-quality forage, exacerbating competition between grain and forage production for limited arable land. These constraints collectively limit the sustainable development and productivity of rice-based cropping systems in Sichuan [20]. To address these challenges, a novel dual-purpose grain and forage production system (Figure 1B), in which the first-season rice crop is harvested as whole-crop silage and the ratoon crop is used for grain production, has recently been proposed [21]. This system aims to achieve multiple objectives, including reduced Cd accumulation, improved grain quality, dual utilization of farmland, and coordinated production of grain and forage. Moreover, harvesting the first-season crop approximately 20 days earlier effectively extends the growing period of the ratoon crop, thereby improving the utilization of thermal and radiation resources and providing a promising alternative for regions such as the western Sichuan Plain, where heat resources are relatively limited [22,23,24].

However, compared with conventional ratoon rice production, the dual-purpose grain and forage production system demands superior cultivar performance. Suitable cultivars should possess strong ratooning ability and a relatively short growth duration while simultaneously maintaining high silage rice yield and quality, high ratoon rice yield and quality, and low Cd accumulation to achieve the coordinated goals of forage and grain production, high productivity, superior quality, and food safety. Previous studies have mainly focused on high-yield cultivation techniques, physiological mechanisms, and low Cd accumulation in ratoon rice, whereas the evaluation and selection of rice cultivars specifically adapted to the dual-purpose grain and forage production system remain largely unexplored. Consequently, the selection criteria and evaluation system for suitable cultivars have yet to be established.

Therefore, the objectives of this study were to systematically evaluate the growth duration, ratooning ability, silage rice yield and quality of the first-season crop, ratoon rice yield of the second-season crop, and Cd accumulation characteristics of 25 widely cultivated rice cultivars under the “first-season forage + second-season ratoon rice” (noted as FR) system, the “first-season middle rice + second-season ratoon rice” (noted as MR) system and the “single middle-season rice” (noted as SMR) system (Figure 1A), respectively, in the southwest regions of China. Principal component analysis was further employed to comprehensively assess cultivar performance and identify elite cultivars suitable for this production system, thereby providing valuable resources and a theoretical basis for cultivar selection, cultivation optimization, and the large-scale application of the dual-purpose grain and forage production system.

## 2. Results

### 2.1. Growth Period

The growth duration of the single middle-season rice among the 25 cultivars ranged from 155 to 163 days, with a maximum difference of 8 days between the earliest- and latest-maturing cultivars (Table 1). The growth duration of the ratoon rice varied from 63 to 103 days, exhibiting a much larger inter-cultivar variation of 40 days. Consequently, the total growth duration of the FR system ranged from 199 to 239 days, with a maximum difference of 40 days among cultivars.

Among the tested cultivars, CKYSM exhibited the longest growth duration for the single middle-season rice (163 days), whereas its ratoon rice matured the fastest, requiring only 63 days, which was identical to TY068. As a result, both cultivars had the shortest total growth duration (199 days) under the FR system. In contrast, FY498, TXYZ, LJY1706, and N5YYX1H showed relatively moderate growth durations for the single middle-season rice (155–157 days), while their ratoon rice required only 71 days, second only to CKYSM. Consequently, these cultivars also completed the FR system within 199 days, the same as CKYSM.

### 2.2. Ratooning Ability

**Figure 2:**
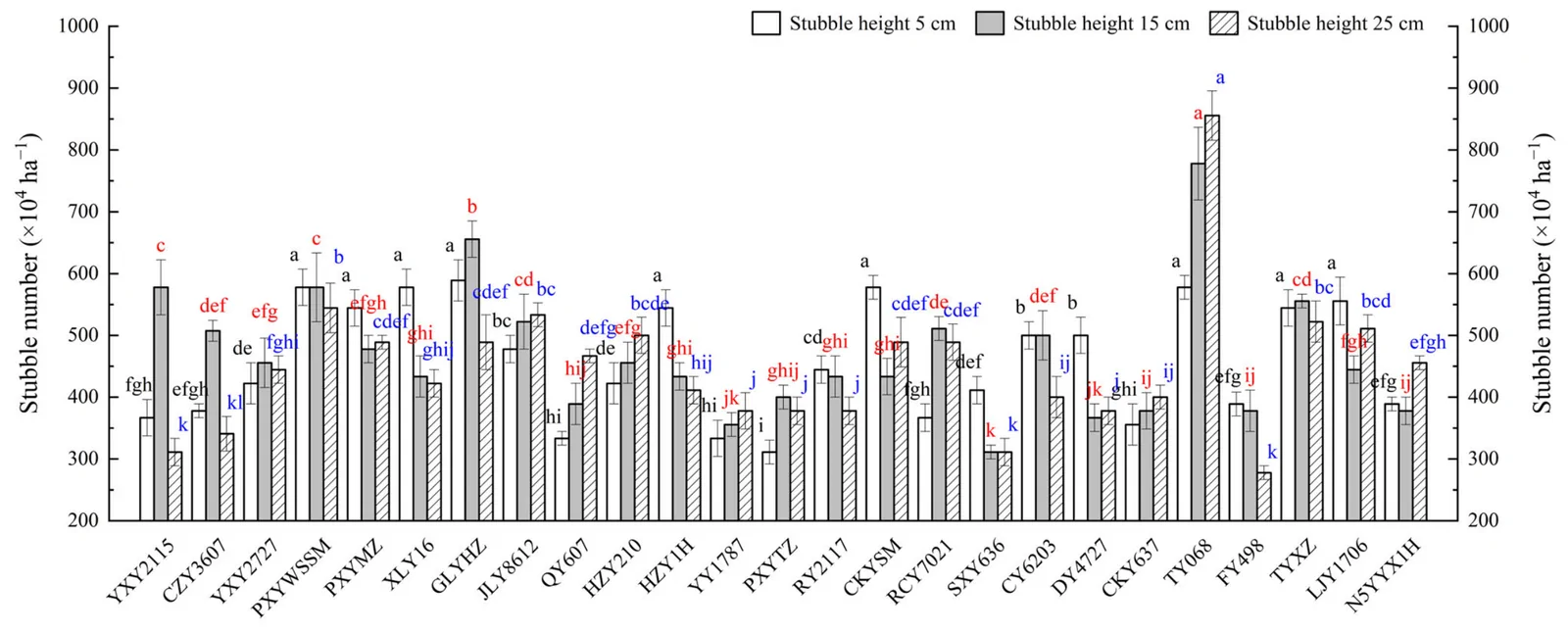
Stubble number of different cultivars under forage-grain dual-purpose ratoon rice system. Different lowercase letters in the same color column indicate significant differences among treatments at *p* < 0.05.

**Figure 3:**
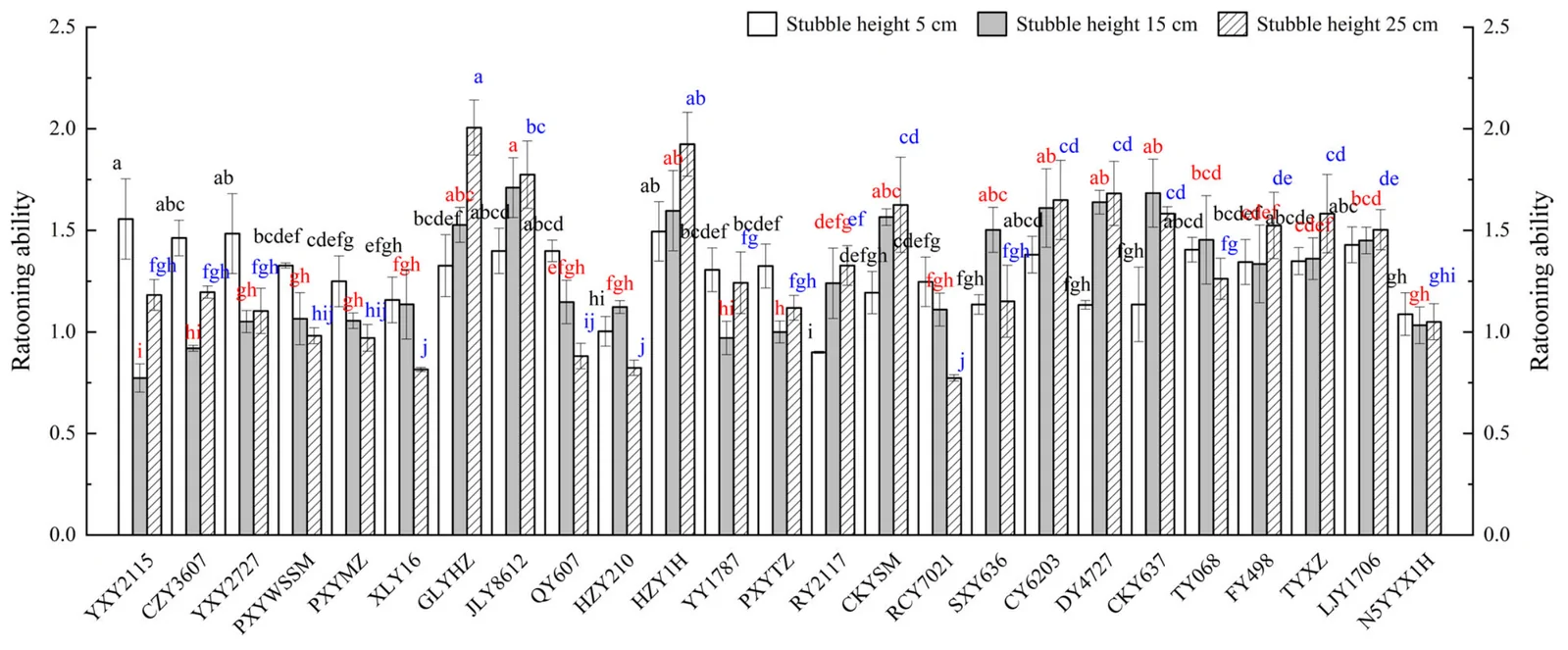
Ratooning ability of different cultivars under forage-grain dual-purpose ratoon rice system. Different lowercase letters in the same color column indicate significant differences among treatments at *p* < 0.05.

**Figure 4:**
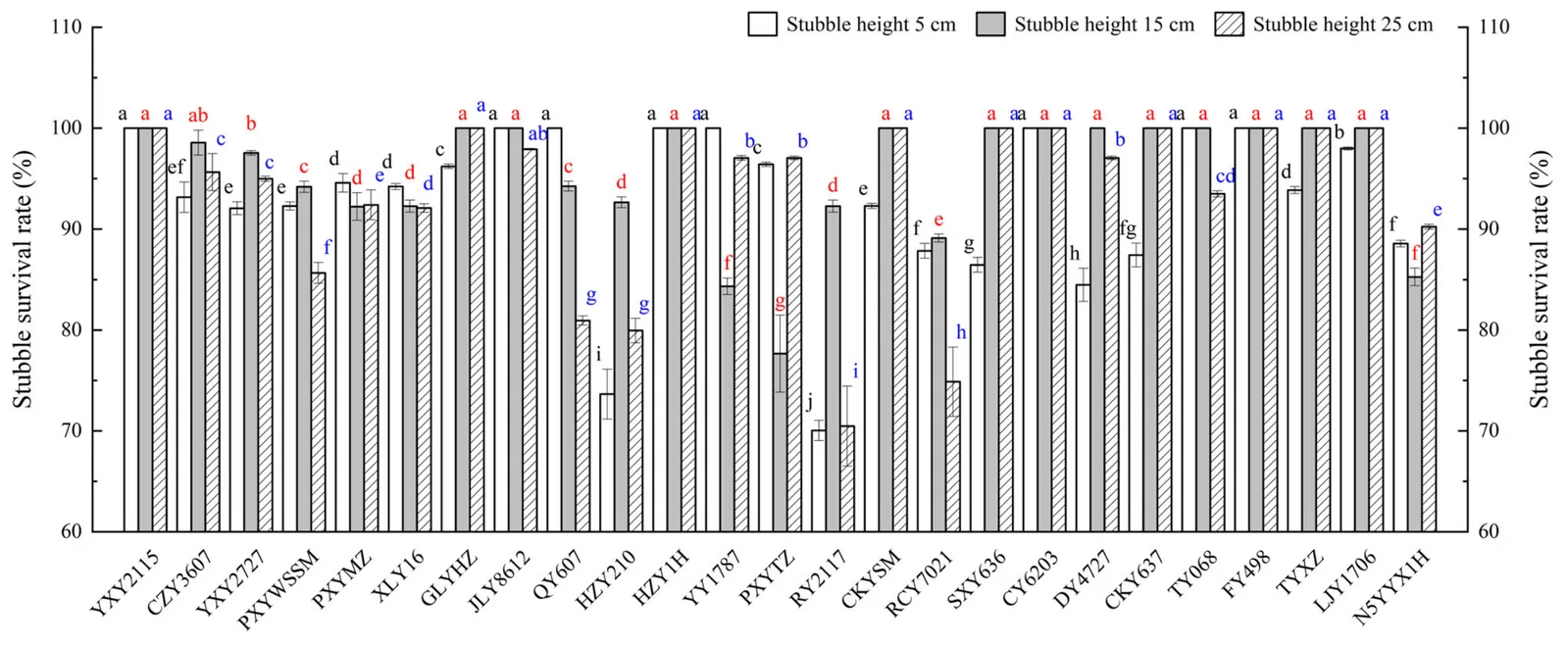
Stubble survival rate of different cultivars under forage-grain dual-purpose ratoon rice system. Different lowercase letters in the same color column indicate significant differences among treatments at *p* < 0.05.

The stubble number of the 25 rice cultivars under the FR system ranged from 311.11 to 855.56 × 10^4^ ha^−1^ (Figure 2), while the ratooning ability (regrowth buds per stubble) varied from 0.77 to 2.01 (Figure 3), and the stubble survival rate ranged from 73.64% to 100.00% (Figure 4). (The complete data relating to stubble number, ratooning ability and stubble survival rate is available in Supplementary Tables S3–S5) Significant differences in both ratooning ability and stubble survival rate were observed among cultivars and across different stubble height treatments. Overall, HZY1H consistently exhibited strong ratooning ability (1.49–1.92) and maintained a 100% stubble survival rate under all stubble height treatments. Similarly, CY6203 and FY498 maintained a stubble survival rate of 100% across all treatments.

Cultivar performance differed markedly under different stubble heights. At a stubble height of 5 cm, YXY2115, CZY3607, YXY2727, CY6203, TY068, TYXZ, and LJY1706 exhibited relatively high ratooning ability (1.35–1.56). Among these cultivars, JLY8612, QY607, YY1787, and TY068 achieved a 100% stubble survival rate. At a stubble height of 15 cm, GLYHZ, JLY8612, CKYSM, SXY636, CY6203, DY4727, and CKY637 showed superior ratooning ability (1.53–1.71), with all maintaining a 100% stubble survival rate. At a stubble height treatment of 25 cm, GLYHZ performed best, exhibiting the highest ratooning ability (2.01) while maintaining a 100% stubble survival rate. In addition, YXY2115, CKYSM, SXY636, CY6203, CKY637, TYXZ, and LJY1706 achieved 100% stubble survival under this treatment.

### 2.3. Yield and Yield Components

Significant differences in grain yield and its yield components were observed among rice cultivars and cultivation systems. Under the SMR system, grain yield ranged from 8.03 to 10.58 t ha^−1^ (Table 2). Despite the low temperature and limited solar radiation at the experimental site, all 25 cultivars (100%) successfully regenerated after being harvested as forage in the first season and completed grain filling and maturation in the ratoon season, producing a harvestable grain yield under the FR system, with ratoon rice yields ranging from 2.23 to 11.00 t ha^−1^. Overall, most cultivars produced higher ratoon rice yields under this system than under the MR system, although yields remained lower than those of the SMR system, with reductions ranging from 6.25% to 76.14%. In contrast, grain filling of the ratoon rice was severely restricted under the MR system. Among the 25 cultivars, 11 failed to achieve normal grain filling, while the remaining 14 cultivars produced significantly lower ratoon rice yields of 2.29–7.08 t ha^−1^, representing yield reductions of 20.74–75.03% relative to the SMR system.

Analysis of the yield components revealed that, compared with the SMR system, both the FR system and MR system generally increased the panicle number, whereas spikelets per panicle and the seed-setting rate were markedly reduced. Specifically, under the FR system, the panicle number increased by an average of 32.63%, while spikelets per panicle and the seed-setting rate decreased by 52.38% and 20.56%, respectively. Under the MR system, the panicle number increased by 15.35% on average relative to the SMR system, whereas spikelets per panicle and the seed-setting rate decreased by 52.21% and 31.18%, respectively.

Overall, the yield advantage of the FR model and high-yielding cultivars over the SMR model and low-yielding cultivars was primarily attributable to stronger ratooning ability, as reflected by greater regeneration of productive tillers and a relatively high seed-setting rate during the ratoon season.

### 2.4. Grain Cd Concentration

**Figure 5:**
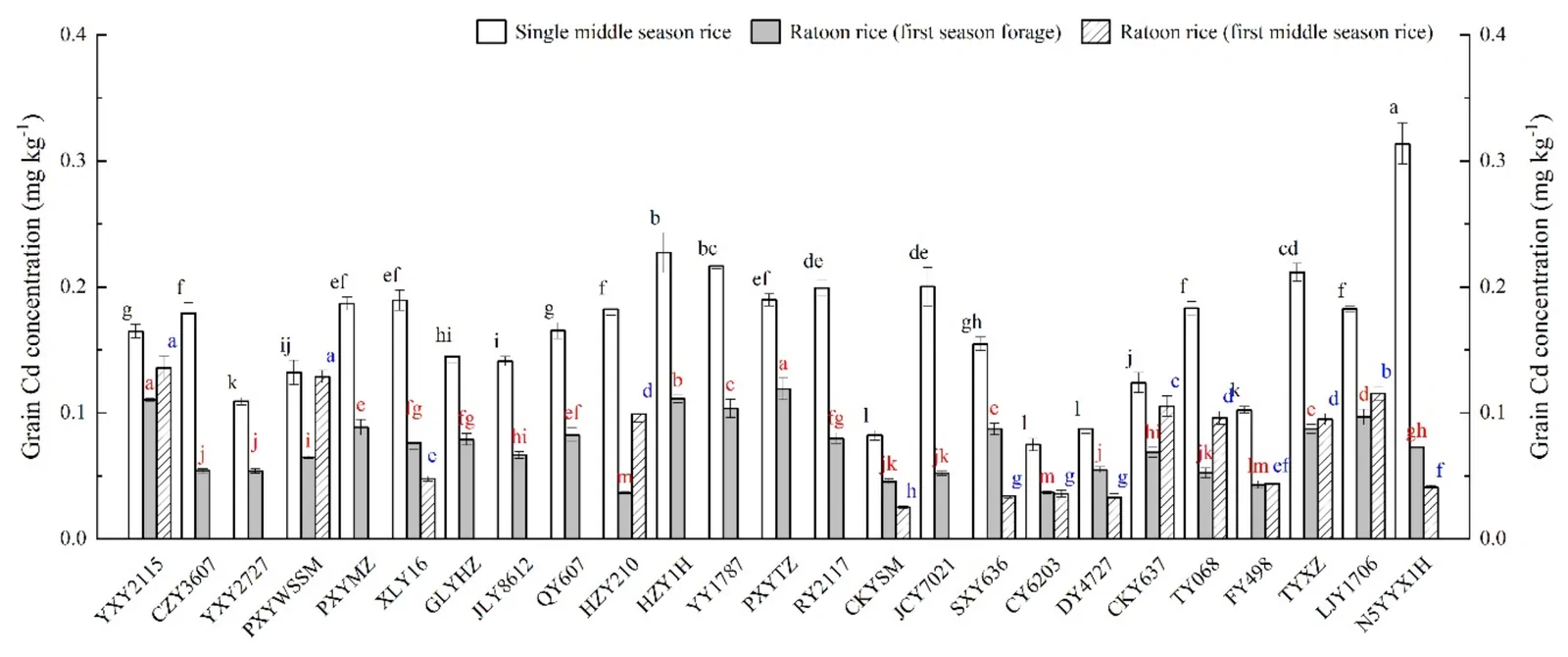
Grain Cd concentrations of different cultivars under different cultivation system. Different lowercase letters in the same color column indicate significant differences among treatments at *p* < 0.05.

**Figure 6:**
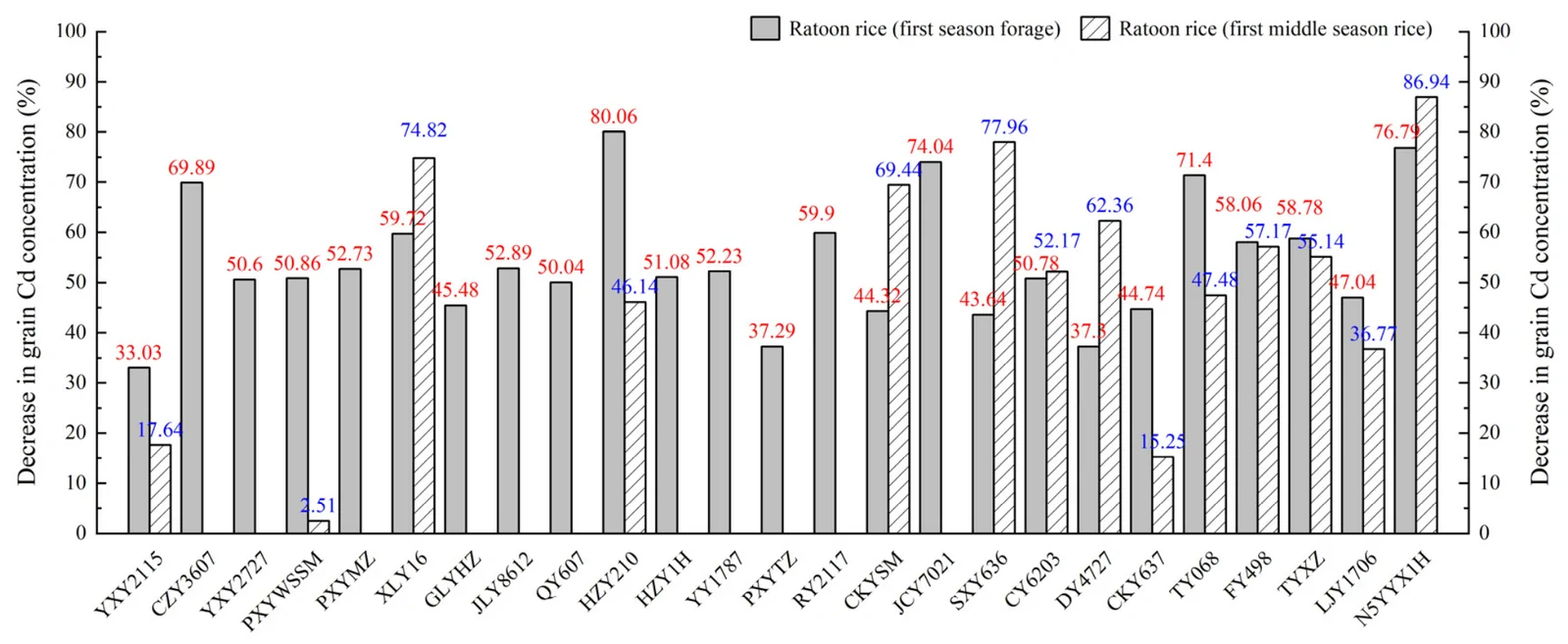
Decrease in Grain Cd concentrations of different cultivars under different cultivation system.

Preliminary soil analyses showed that the experimental field contained 0.3780 mg kg^−1^ of available Cd, with a soil pH of 6.75, indicating slightly acidic conditions. Grain Cd concentrations differed significantly among rice cultivars and cultivation systems (Figure 5 and Figure 6).

Under the SMR system, grain Cd concentrations ranged from 0.0820 to 0.3138 mg kg^−1^, with N5YYX1H exhibiting the highest value and CY6203 the lowest. Under the FR system, grain Cd concentrations ranged from 0.0364 to 0.1190 mg kg^−1^, with the highest concentration observed in PXYTZ and the lowest in HZY210. Under the MR system, grain Cd concentrations ranged from 0.0251 to 0.1359 mg kg^−1^, with the highest and lowest values recorded in YXY2115 and CKYSM, respectively.

Overall, both ratoon rice systems exhibited substantially lower grain Cd concentrations than the SMR system. Under the FR system, grain Cd concentrations decreased by 33.03–80.06% across cultivars, with an average reduction of 54.11% relative to the SMR system. Similarly, under the MR system, grain Cd concentrations were reduced by 2.51–86.94%, with an average decrease of 50.13%.

Further comparison revealed cultivar-specific differences in Cd reduction between the two ratoon rice systems. For YXY2115, PXYWSSM, HZY210, CKY637, TY068, FY498, TYXZ, and LJY1706, the reduction in grain Cd concentrations was smaller under the MR system than under the FR system. In contrast, XLY16, CKYSM, SXY636, CY6203, DY4727, and N5YYX1H exhibited the opposite trend, showing greater reductions in grain Cd concentrations under the MR system.

### 2.5. Silage Rice Yield

**Figure 7:**
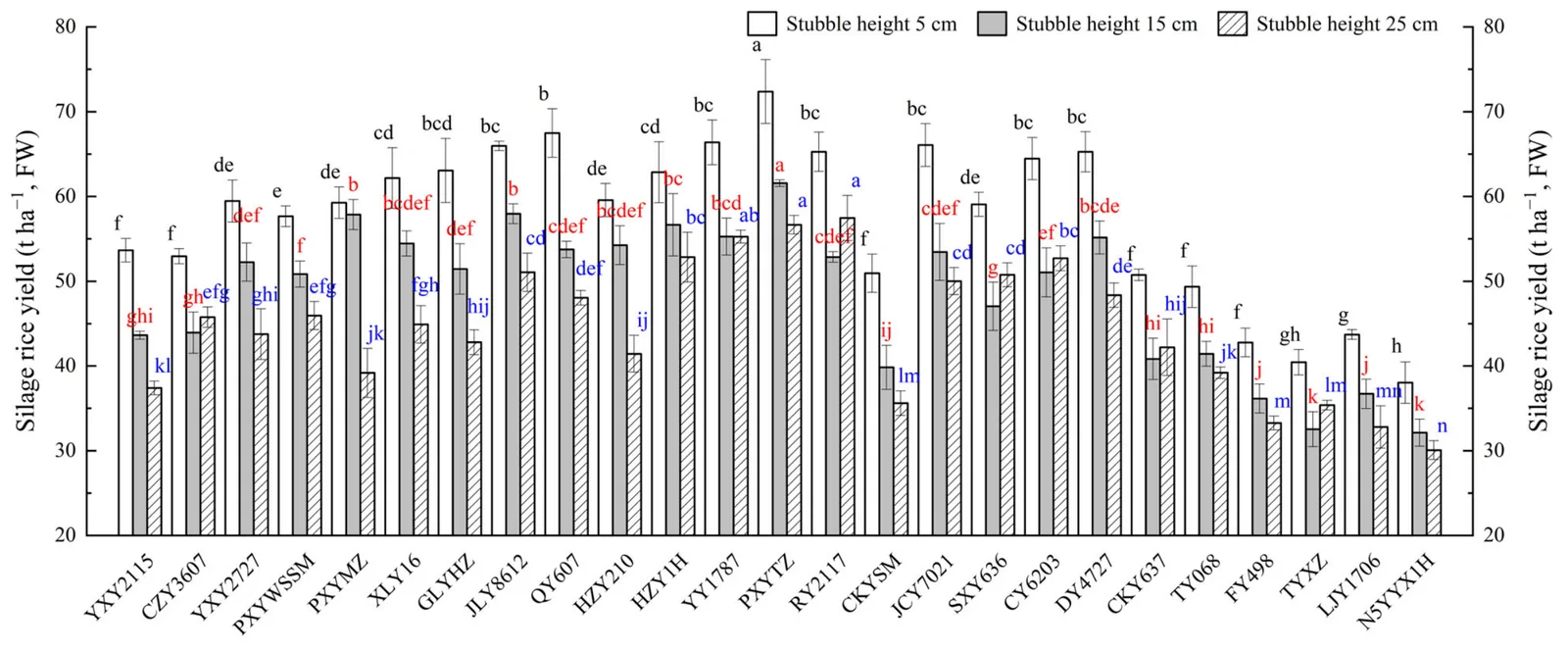
Silage rice yield of different cultivars under forage-grain dual-purpose ratoon rice system (fresh weight). FW represents fresh weight. Different lowercase letters in the same color column indicate significant differences among treatments at *p* < 0.05.

**Figure 8:**
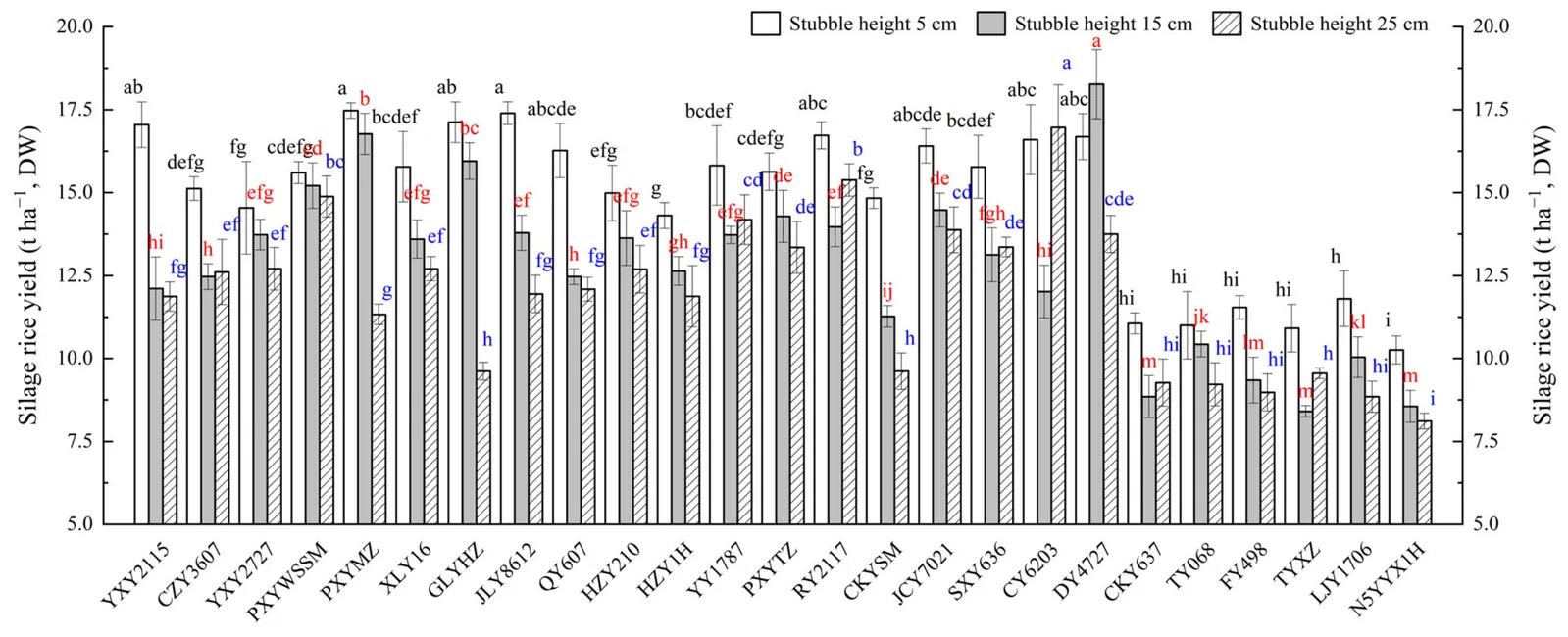
Silage rice yield of different cultivars under forage-grain dual-purpose ratoon rice system (dry weight). DW represents dry weight. Different lowercase letters in the same color column indicate significant differences among treatments at *p* < 0.05.

Both rice cultivar and stubble height significantly affected the fresh weight (FW) and dry weight (DW) of silage rice (Figure 7 and Figure 8). Overall, both FW and DW declined with increasing stubble height. At a stubble height of 5 cm, forage DW ranged from 10.26 to 17.47 t ha^−1^. At 15 cm, it ranged from 8.41 to 18.27 t ha^−1^, whereas at 25 cm, it ranged from 8.11 to 16.96 t ha^−1^. Compared with the 5 cm treatment, the average DW decreased by 13.90% and 19.40% under the 15 cm and 25 cm stubble height treatments, respectively.

Although PXYTZ consistently produced relatively high FW across all stubble heights (56.66–72.37 t ha^−1^), its DW remained comparatively low (13.35–15.63 t ha^−1^). The cultivar with the highest DW varied among stubble height treatments. At a 5 cm stubble height, PXYMZ produced the highest DW (17.47 t ha^−1^). At 15 cm, the highest value was recorded for DY4727 (18.27 t ha^−1^), whereas CY6203 achieved the greatest DW (16.96 t ha^−1^) under the 25 cm stubble height treatment.

### 2.6. Silage Rice Quality

Silage rice quality is comprehensively determined by the concentrations of crude protein (CP), ADF, NDF, and starch. In general, higher concentrations of CP and starch together with lower concentrations of ADF and NDF indicate superior silage rice quality. Based on the preliminary evaluation, 11 rice cultivars that have been widely adopted in commercial production were selected for a comprehensive assessment and comparison of silage rice quality.

According to the Chinese National Standard for silage maize quality classification (GB/T 25882-2010), the CP concentration of silage rice from all 11 cultivars met the Grade I standard (≥7%), indicating a high protein nutritional value (Table 3). Except for CY6203, CKY637, TYXZ, LJY1706, and N5YYX1H, the remaining seven cultivars satisfied the Grade III standard for starch concentration (≥15%), suggesting a favorable energy supply potential. In terms of ADF concentration, YXY2115, CKYSM, XLY16, DY4727, and FY498 met the Grade III or higher standard (≤29%), indicating relatively low cell wall fiber content and consequently better digestibility. Regarding NDF concentration, only CKY637 failed to meet the minimum Grade III standard (≤55%), implying comparatively low silage digestibility and palatability.

RFV, calculated from the silage rice quality components, further demonstrated substantial differences among cultivars. XLY16 exhibited the highest RFV, whereas CKY637 had the lowest, indicating that XLY16 possessed the best overall silage rice quality and feeding value among the tested cultivars, while CKY637 showed comparatively inferior silage performance.

### 2.7. Principal Component Analysis

To comprehensively and objectively evaluate the suitability of different cultivars for the dual-purpose grain and forage production system, assessment based on a single trait is insufficient to capture their overall performance. Therefore, principal component analysis (PCA) was conducted using five key indicators: silage rice yield (15 cm stubble height), grain Cd concentration, RFV, ratooning ability (15 cm stubble height), and ratoon rice yield.

CKY637 obtained the highest comprehensive score and ranked first among the tested cultivars (Table 4), mainly due to its strong ratooning ability (1.61), relatively high ratoon rice yield (8.13 t ha^−1^), and favorable performance in Cd accumulation (0.0686 mg kg^−1^). Although CKY637 had the highest comprehensive score, it showed relatively low silage rice yield (8.85 t ha^−1^) and the lowest RFV (77.08); therefore, CKY637 should not be considered an “overall optimal” cultivar but rather a candidate cultivar with specific advantages. In contrast, YXY2115 ranked last despite having relatively high silage rice yield (12.11 t ha^−1^) and RFV (188.36). Its inferior ranking was primarily associated with lower ratooning ability (0.77), lower ratoon rice yield (6.78 t ha^−1^), and the highest grain Cd concentration (0.1105 mg kg^−1^) among the tested cultivars.

Among the higher-ranked cultivars, CY6203 and LJY1706 exhibited favorable silage rice yield (12.02 and 10.04 t ha^−1^, respectively) and RFV (108.45 and 112.60, respectively). However, CY6203 produced a comparatively low ratoon rice yield (7.47 t ha^−1^), whereas LJY1706 exhibited a relatively high grain Cd concentration (0.0967 mg kg^−1^). By contrast, CKYSM and FY498 displayed a more balanced performance across all evaluated traits, including silage rice yield (11.27 and 9.34 t ha^−1^), grain Cd concentration (0.0457 and 0.0430 mg kg^−1^), RFV (118.21 and 119.34), ratooning ability (1.57 and 1.33), and ratoon rice yield (7.83 and 9.65 t ha^−1^, respectively).

These results suggest that CKYSM and FY498 achieved a more favorable balance among silage rice productivity, quality, grain Cd concentration, ratooning ability, and ratoon rice yield than the other tested cultivars. Accordingly, these two cultivars may serve as suitable genetic materials for future studies aimed at optimizing cultivation practices for the dual-purpose grain and forage production system.

## 3. Discussion

### 3.1. Effect of Growth Duration on Ratoon Rice Performance

The success of ratoon rice production largely depends on the availability of favorable thermal conditions during heading and grain filling [25]. In southwest China, the ratoon crop generally heads and fills grain from mid-September to mid-October, coinciding with the rainy season in western China, when fluctuating temperatures and insufficient heat frequently reduce seed setting and grain filling, thereby limiting ratoon rice productivity [26,27,28]. Although recent climate warming has extended the safe growing season and promoted the northward expansion of ratoon rice cultivation in the Sichuan Basin, the increasing occurrence of extreme weather events has increased the uncertainty of thermal resources, posing new challenges for stable production [2,29]. The three cultivation systems examined in this study differ in the utilization of the first-season crop and the second-season crop. While the SMR system produces grain from a single-season crop, the MR and FR systems both rely on ratoon rice production but differ in the use of the first-season crop, with the former producing grain and the latter producing forage. Thus, compared with the SMR and MR systems, the FR system places additional emphasis on the cultivar’s ability to regenerate after the forage harvest while still completing the subsequent ratoon grain production cycle.

**Figure 9:**
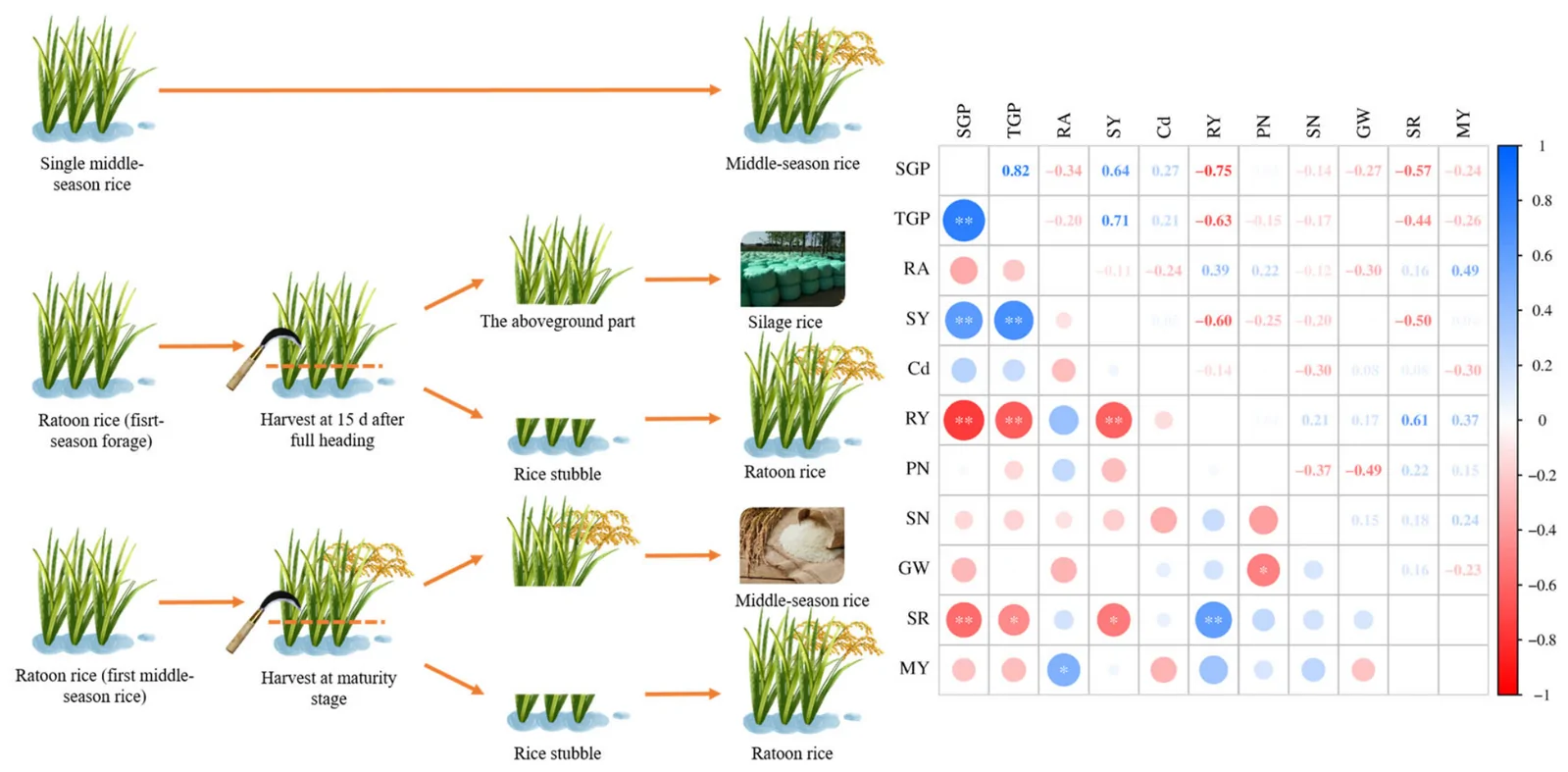
Diagram illustrating the different cultivation systems and correlation coefficients among the main indexes. SGP, TGP, RA, SY, Cd, RY, PN, SN, GW, SR and MY represent silage rice growth period, total growth period, ratooning ability, silage rice yield, grain Cd concentration, ratoon rice yield, panicle number, spikelet number per panicle, 1000-grain weight, seed-setting rate and single middle-season rice yield, respectively. ANOVA *p* values and symbols were defined as: * *p* < 0.05; ** *p* < 0.01.

Growth duration is therefore a critical determinant of cultivar adaptation to the dual-purpose grain and forage production system. Previous studies have demonstrated that ratooning ability is a quantitatively inherited trait that varies considerably among genotypes and is strongly influenced by environmental conditions and crop management [30,31]. Consistent with these findings, our results showed significant genotypic variation in both growth duration and ratooning ability [32,33]. Correlation analysis further revealed that the growth duration of the silage rice was negatively correlated with ratooning ability (r = −0.336), whereas ratooning ability was positively correlated with the ratoon rice yield (r = 0.389), panicle number (r = 0.225), and seed-setting rate (r = 0.164) (Figure 9). These results suggest that shortening the growth duration of silage rice may enhance ratoon regeneration and ultimately improve ratoon rice productivity.

The contrasting performance of cultivars under the two production systems further highlights the importance of growth duration. Under the MR system, 11 of the 25 cultivars failed to complete normal grain filling, whereas all cultivars successfully produced a ratoon crop under the FR system. Six cultivars, including CKYSM, TY068, FY498, TYXZ, LJY1706, and N5YYX1H, completed the entire production cycle within 200 days, while the remaining cultivars required approximately 230 days. Moreover, the total growth duration of the MR system was negatively correlated with the ratoon rice yield (r = −0.626), indicating that delayed crop development increased the risk of exposing the ratoon crop to unfavorable low-temperature conditions during reproductive growth.

These findings emphasize that cultivar selection is fundamental to the successful adoption of the dual-purpose grain and forage production system [34,35]. Under the climatic conditions of Mianzhu, where the average temperature declined rapidly approximately 200 days after sowing (Figure S1), cultivars with a total growth duration of ≤200 days are more likely to complete heading and grain filling within the safe thermal window. Therefore, priority should be given to cultivars combining a short growth duration with strong ratooning ability to maximize both forage and ratoon grain production while minimizing the risk of low-temperature damage.

### 3.2. Relationship Between Ratoon Rice Yield and Yield Components

Improving rice productivity remains a fundamental strategy for ensuring food security in China. As opportunities to further expand the rice cultivation area become increasingly limited, increasing the cropping index through ratoon rice cultivation provides an effective approach to enhancing grain production without additional land input [36]. The three cultivation systems evaluated in this study differ in their utilization of the first-season crop, with the SMR system producing grain from a single middle-season crop, the MR system producing grain from both first-season and second-season crops, and the FR system producing forage from the first-season crop followed by ratoon grain production. These differences in crop utilization may impose distinct requirements on yield formation and cultivar adaptation, particularly for ratoon rice production under the FR system. Ratoon rice yield is determined by the combined effects of the panicle number, spikelets per panicle, the seed-setting rate, and the 1000-grain weight [37,38]. Previous studies have generally identified the panicle number as the dominant yield component, followed by spikelets per panicle, the seed-setting rate, and the 1000-grain weight [39,40].

Consistent with previous reports, significant differences in grain yield were observed among cultivars and cultivation systems in the present study. However, correlation analysis indicated that, under the FR system, grain yield was most strongly associated with the seed-setting rate (r = 0.610), whereas its correlations with the panicle number (r = 0.037), spikelets per panicle (r = 0.211), and the 1000-grain weight (r = 0.165) were comparatively low (Figure 9). These results suggest that maintaining a high seed-setting rate, rather than simply increasing the panicle number, was the primary determinant of high ratoon rice yield under the conditions of this study.

Previous studies have reported that the panicle number and seed-setting rate are often negatively correlated and that the relative contribution of individual yield components varies with the genotype and growing environment [41,42]. In the present study, high-yielding cultivars under the FR system achieved superior productivity primarily through a higher seed-setting rate, accompanied by relatively favorable spikelets per panicle and 1000-grain weight. This differs from previous studies in which the panicle number was considered the dominant contributor to ratoon rice yield [43], suggesting that the yield-limiting factors may shift under different cultivation objectives and production systems.

A significant trade-off was also observed between first-season silage rice yield and second-season ratoon rice yield. The two traits were significantly negatively correlated (r = −0.600) (Figure 9), indicating that cultivars with higher silage rice yields generally produced lower ratoon rice yields, whereas cultivars with stronger ratooning ability tended to produce lower silage rice yields. This trade-off is likely attributable to competition for assimilates between vegetative biomass accumulation in the first-season crop and the development of ratoon buds. Therefore, cultivar selection for the dual-purpose grain and forage production system should aim to optimize the balance between forage production and ratoon rice yield rather than maximizing either trait alone.

Ratooning ability was closely associated with yield formation but did not directly determine ratoon rice yield. Consistent with previous findings [44], ratooning ability was positively correlated with ratoon rice yield, panicle number, and seed-setting rate but negatively correlated with spikelets per panicle and 1000-grain weight. These relationships further highlight the importance of assimilate allocation and ratoon bud vigor during yield formation and suggest that cultivars combining strong ratooning ability with a high seed-setting rate are more suitable for achieving stable and high productivity in the dual-purpose grain and forage production system.

### 3.3. Grain Cd Concentration and Implications for the Safe Utilization of Cd-Contaminated Paddy Fields

Cd is one of the most hazardous heavy metals in paddy soils because rice readily absorbs and accumulates Cd, making rice grains a major dietary source of Cd exposure in many rice-consuming regions [45,46]. Among the available mitigation strategies, the use of low-Cd-accumulating cultivars combined with appropriate agronomic management is considered one of the most practical and cost-effective approaches for reducing grain Cd concentrations [11,12]. Consistent with previous studies, significant differences in grain Cd concentrations were observed among the tested cultivars (Figure 6 and Figure 7). Although most cultivars met the Chinese food safety limit (<0.20 mg kg^−1^), several cultivars exhibited Cd concentrations close to or exceeding this threshold, highlighting substantial variation in Cd accumulation capacity under the same soil conditions [47]. These results further demonstrate that cultivar selection plays a critical role in the safe utilization of Cd-contaminated paddy fields. The three cultivation systems differed in their production objectives and Cd accumulation patterns. Compared with the SMR system and MR system, the FR system combines first-season forage production with subsequent ratoon grain production while exhibiting a substantially lower grain Cd concentration in the ratoon crop. This system-level difference provides an important context for further evaluating the performance of individual cultivars.

Previous studies have shown that Cd is preferentially retained in vegetative tissues rather than reproductive organs and that its distribution differs between the first-season and ratoon crops [48]. In the present study, the grain Cd concentration was consistently lower in the ratoon crop than in the first-season crop, with an average reduction of 54.11% under the FR system. This reduction is likely attributable to the harvest of aboveground biomass 15 days after heading, which removed a considerable proportion of Cd before grain filling was completed, thereby reducing Cd translocation from vegetative tissues to developing grains. Similar reductions in heavy metal accumulation during regrowth have been reported in other perennial or ratooning crops [15,16,17], suggesting that regrowth may alter the uptake, redistribution, and partitioning of mineral elements.

From a practical perspective, the dual-purpose grain and forage production system provides a promising strategy for the safe utilization of Cd-contaminated paddy fields. First, harvesting the first-season crop as forage removes part of the Cd accumulated in the aboveground biomass, thereby reducing the risk of subsequent Cd translocation to ratoon grains. Second, the markedly lower grain Cd concentration in the ratoon crop increases the probability of meeting food safety standards. Finally, the system simultaneously produces forage and grain, improving land-use efficiency while supporting the coordinated development of crop and livestock production. These advantages are consistent with current strategies for sustainable agriculture and the safe utilization of contaminated farmland [49,50].

Correlation analysis further showed a negative relationship between the grain Cd concentration and ratoon rice yield (r = −0.138) (Figure 9), indicating that lower Cd accumulation did not compromise yield potential. Therefore, cultivar selection in Cd-contaminated areas should simultaneously consider grain yield and Cd accumulation to identify cultivars capable of achieving both high productivity and low Cd concentrations. These findings suggest that the reduction in grain Cd concentrations achieved by the dual-purpose grain and forage production system results not only from cultivar differences but also from a redistribution of Cd within the plant caused by early biomass removal, providing a novel agronomic strategy for the safe utilization of Cd-contaminated paddy fields.

Nevertheless, the Cd concentration in the first-season forage biomass was not determined in the present study. Therefore, although the present results demonstrate the potential advantage of the dual-purpose grain and forage production system in reducing Cd accumulation in ratoon rice grain, the safety of the harvested forage for animal feed cannot be fully evaluated. Future studies should simultaneously assess Cd accumulation in forage biomass and its transfer along the feed–animal product pathway to comprehensively evaluate the safety of this production system.

### 3.4. Characteristics of Cultivars Suitable for the Dual-Purpose Grain and Forage Production System

The present study indicates that rice cultivars suitable for the dual-purpose grain and forage production system should possess a combination of agronomic, physiological, and quality-related characteristics to maximize both forage and grain production. First, a short total growth duration is essential to ensure timely crop rotation and efficient utilization of available thermal and solar resources. For example, cultivars such as CKYSM and FY498, with a total growth duration of approximately 199 days, exhibited excellent adaptability to the production system.

Second, ratooning ability is a key determinant of cultivar suitability. Cultivars with strong ratooning ability are capable of maintaining high stubble survival and vigorous ratoon tiller regeneration under different stubble height treatments. In the present study, HZY1H and CY6203 consistently exhibited superior ratooning performance across a range of stubble heights, indicating their strong regenerative potential.

Grain productivity should also be evaluated by considering both forage production in the first season and grain yield of the ratoon crop. Among the tested cultivars, LJY1706 produced a ratoon rice yield of 11.00 t ha^−1^ under the FR system, representing a 6.25% increase compared with the SMR system. This superior yield performance was primarily associated with its greater ratoon tillering capacity and relatively high seed-setting rate.

In addition to grain productivity, silage rice yield and quality are critical selection criteria. Although increasing the stubble height generally reduced forage biomass, cultivars such as DY4727 and CY6203 maintained relatively high DW yields even under higher stubble height treatments. Regarding silage rice quality, all tested cultivars exhibited high CP concentrations, whereas substantial differences were observed in starch and fiber components. Notably, XLY16 achieved the highest RFV, indicating superior overall silage rice quality.

Taken together, the results suggest that an ideal cultivar for the dual-purpose grain and forage production system include the following characteristics: (1) a total growth duration of ≤ 200 days; (2) ratooning ability > 1.5; (3) a stubble survival rate approaching 100%; (4) ratoon rice yield of ≥ 9.65 t ha^−1^ under the first-season forage and second-season ratoon rice system; (5) a reduction in grain Cd concentrations of > 50%, resulting in a ratoon rice Cd concentration of < 0.05 mg kg^−1^; and (6) a silage rice yield of > 9.0 t ha^−1^ together with an RFV > 115. Achieving an optimal balance among these traits, rather than maximizing any single characteristic, appears to be the key prerequisite for developing rice cultivars suitable for dual-purpose grain and forage production. These indicators may serve as reference criteria for subsequent cultivar screening and evaluation rather than as strict thresholds for cultivar selection.

## 4. Materials and Methods

### 4.1. Experimental Site

This experiment was conducted in 2024 at the experimental base of the Grain-Economic Complex Expert Compound in Mianzhu, Sichuan Province (N 31°15′, E 104°13′). The experiment site comprised loam soil, and the previous crop was a vegetable. Prior to the establishment of the field experiment, soil samples from the topsoil layer (0–0.20 m) were analyzed. The national meteorological monitoring station (CAWS600, China Huayun Meteorological Technology Group Co., Ltd., Beijing, China) was used to record the average daily temperature, maximum temperature and rainfall in the rice-growing season. The complete data relating to the topsoil layer and climate is available in Supplementary Table S1 and Figure S1, respectively. During the rice growth period, the daily average temperature was 23.01 °C, the accumulated temperature above 10 °C reached 5225.11 °C, and the daily average rainfall was 4.76 mm. Twenty-five rice cultivars widely cultivated in the southwest regions of China were selected as experimental materials, most of which had participated in the “Daoxiang Cup” rice quality competition. Detailed characteristics of the tested cultivars are provided in Supplementary Table S2.

### 4.2. Experimental Design

The experiment was arranged in a randomized complete block design with three replicates. Seeds were sown on 15 March 2024, transplanted on 15 April, and the latest-maturing cultivar was harvested on 28 October (first-season forage + second-season ratoon rice system). Rice was established by manually simulating mechanical transplanting with four seedlings per hill, a hill spacing of 12 cm, and a row spacing of 25 cm. The plot area for each cultivar was 30 m^2^. Urea (46.4% N), calcium superphosphate (12.0% P_2_O_5_), and potassium chloride (60.0% K_2_O) were used as the sources of nitrogen (N), phosphorus (P), and potassium (K), respectively.

(1) The “first-season forage + second-season ratoon rice” (noted as FR) system (Figure 1B) and the “first-season middle rice + second-season ratoon rice” (noted as MR) system received the same fertilizer management. A total of 300.0 kg N ha^−1^ was applied to the main crop, with N split between basal, tillering, and panicle fertilizers at a ratio of 3:3:4. Basal fertilizer was applied 1 day before transplanting, tillering fertilizer at 7 days after transplanting, and panicle fertilizer at the fourth leaf from the top (the fourth leaf stage before flag leaf emergence). N, P, and K were applied at an overall ratio of N:P_2_O_5_:K_2_O = 2:1:2. All P fertilizer was applied as basal fertilizer, whereas K fertilizer was split equally, with 50% applied as basal fertilizer and the remaining 50% at the fourth leaf stage.

(2) In the ratoon crop, 75 kg N ha^−1^ and 45 kg K_2_O ha^−1^ were applied 10 days before harvesting the first-season forage or grain. For the forage treatment, the first-season crop was harvested for silage 15 days after full heading. Following harvest and re-irrigation, an additional 75 kg N ha^−1^ and 45 kg K_2_O ha^−1^ were applied. Irrigation was resumed immediately after the removal of the first-season straw to prevent damage to rice stubble caused by high-temperature exposure and to suppress excessive weed emergence resulting from insufficient water depth.

(3) In the “single middle-season rice” (noted as SMR) system, fertilizer was applied at rates of 180.0 kg N ha^−1^, 75.0 kg P_2_O_5_ ha^−1^, and 150.0 kg K_2_O ha^−1^. N was split among basal, tillering, and panicle applications at a ratio of 5:3:2. Basal fertilizer was applied 1 day before transplanting, tillering fertilizer at 7 days after transplanting, and panicle fertilizer at the fourth leaf stage. All P fertilizer was applied as basal fertilizer, while K fertilizer was equally divided between the basal and panicle fertilizer applications. Field management, including the prevention and control of pests and weeds, was conducted according to the local cultural practices.

At the first-season forage harvest, all tested cultivars were subjected to three stubble height treatments (5, 15, and 25 cm). The plants were cut manually at the designated height above the soil surface, and the harvested aboveground biomass was collected for forage yield determination. The remaining stubble was retained in the field to allow ratoon growth. Ratooning ability, stubble survival, and ratoon rice yield were subsequently evaluated under the three stubble height treatments. For the comprehensive evaluation of cultivar performance, the data obtained under the 15 cm stubble height treatment were used for PCA analysis. This treatment was selected based on our preliminary research, which showed that a 15 cm stubble height generally resulted in relatively favorable and balanced performance across the major evaluated traits.

### 4.3. Measurements and Methods

#### 4.3.1. Ratoon Rice Yield and Yield Components

At the maturity stage, 60 hills were randomly selected from each plot to investigate the average tillering number per hill. Subsequently, six representative plants were selected from each plot to determine the number of panicles per plant, spikelets number per panicle, seed-setting rate, and 1000-grain weight. Grain yield was determined separately by harvesting the designated area of each plot and was adjusted to a standard moisture content of 13.5%.

#### 4.3.2. Grain Cd Concentration

At harvest, 10 hills were randomly sampled from each plot and blanched at 110 °C for 30 min, followed by drying at 80 °C to a constant weight. The samples were then weighed, and the brown rice was obtained, ground, and passed through a 100-mesh sieve for Cd concentration analysis. All grain Cd concentrations reported in this study were determined based on brown rice samples. For Cd analysis, 0.200 g of the ground sample was transferred to a 50 mL conical flask, followed by the addition of 10.0 mL of a mixed acid solution (V_HNO3_/V_HClO4_ = 4:1). The mixture was allowed to stand overnight and was subsequently digested at 220 °C until the solution became clear. When the solution was nearly evaporated to dryness, digestion was terminated, and the flask was allowed to cool naturally. The residue was rinsed with ultrapure water and quantitatively transferred to a 50 mL volumetric flask, where the solution was brought to volume with ultrapure water. The resulting solution was then filtered through a 0.45 μm membrane into a 10 mL centrifuge tube. The Cd concentration in the filtrate was determined using inductively coupled plasma mass spectrometry (ICP-MS; Agilent 7700x, Agilent Technologies Co., Ltd., Santa Clara, CA, USA).

#### 4.3.3. Silage Rice Yield

Fifteen days after full heading, the border rows of each plot were removed, and all aboveground biomass within the plots was harvested to determine fresh forage yield.

#### 4.3.4. Silage Rice Quality

Silage rice quality was not evaluated for all 25 cultivars. The 11 cultivars that failed to complete normal grain filling and maturation in the second-season rice crop under the MR system were first excluded. Among the remaining 14 cultivars, the 11 cultivars with the largest approximate promotion areas were selected for silage rice quality analysis.

At the time of forage harvest, 10 representative plants were selected from each plot. The plant samples were cut into segments approximately 2 cm in length. Stems, leaves, and panicles were thoroughly mixed and packed into silage bags, vacuum sealed, and allowed to ferment naturally at room temperature for 60 d. After fermentation, the quality parameters of the silage were analyzed. Silage rice quality assessment was conducted by Hangzhou Aiko Testing Technology Co., Ltd. Evaluation of first-season silage rice quality followed the national standard GB/T 25882-2010 of the People’s Republic of China, Classification of Silage Corn Quality. Relative feed value (RFV) was calculated using the following equations:

Dry matter intake (%) = 120/Neutral detergent fiber (NDF) content

Digestible dry matter (%) = 88.9 − 0.779 × Acid detergent fiber (ADF) content

Relative feed value = (Dry matter intake × Digestible dry matter)/1.29

#### 4.3.5. Ratooning Ability

The capacity of rice stubble to produce regrowth buds after harvesting the first-season rice was calculated as the number of regrowth buds per rice stubble:

Ratooning ability = Number of regrowth buds/Number of rice stubbles

### 4.4. Statistical Analysis

Data were analyzed using analysis of variance (ANOVA) after checking the assumptions of homogeneity of variances and normality, and means were compared based on the least significant difference (LSD) test at the 0.05 probability level using SPSS 21.0 (Statistical Product and Service Solutions Inc., Chicago, IL, USA). Origin Pro 2022 (OriginLab, Northampton, MA, USA) was used to do the correlation analysis and draw the figures. Principal component analysis of five indexes—silage rice yield (15 cm stubble height), grain Cd concentration, RFV, ratooning ability (15 cm stubble height), and ratoon rice yield—among the different treatments was used to establish a comprehensive evaluation model.

Before PCA, the five selected indexes were processed according to their positive or negative effects, with four positive indexes and one negative index (grain Cd concentration). The processed data were subsequently subjected to PCA. Three principal components were retained, accounting for 92.696% of the total variance, with individual variance contribution rates of 47.989%, 32.595%, and 12.111%, respectively. A comprehensive score was calculated as the weighted sum of the three principal component scores, using their respective variance contribution rates relative to the cumulative variance contribution rate as weights. Thus, the comprehensive score was calculated as F = (0.480/0.927) × F_1_ + (0.326/0.927) × F_2_ + (0.121/0.927) × F_3_, where F is the comprehensive score and F_1_–F_3_ are the scores of the three principal components (detailed information is available in Supplementary Tables S6 and S7).

## 5. Conclusions

Significant variation was observed among rice cultivars in terms of ratooning ability, forage and grain productivity, and Cd accumulation under the dual-purpose grain and forage production system. Based on a comprehensive evaluation integrating ratooning ability (0.77–2.01), silage rice yield (10.26–18.27 t ha^−1^), ratoon rice yield (2.23–11.00 t ha^−1^), and grain Cd concentration (0.0364–0.3138 mg kg^−1^), CKY637 (highest comprehensive score but not necessarily the most balanced cultivar), CKYSM (excellent stability in low Cd accumulation), and FY498 (well-balanced yield and quality) showed favorable overall performance under the conditions tested in this study. These cultivars may serve as potential candidates for further evaluation and optimization of the dual-purpose grain and forage production system. However, the present findings were obtained from a single location and one growing season. Multi-location and multi-year validation trials are therefore needed to assess the stability, adaptability, and consistency of cultivar performance and the effectiveness of this production system across different environments.

## Figures and Tables

**Table 1:** Growth period of different cultivars under different cultivation systems.

Cultivar	Growth Period		
	Single Middle-Season Rice	First-Season Silage Rice	Second-Season Ratoon Rice
YXY2115	163	146	85
CZY3607	159	146	93
YXY2727	163	146	93
PXYWSSM	163	146	85
PXYMZ	156	148	91
XLY16	163	148	83
GLYHZ	163	148	91
JLY8612	163	148	91
QY607	159	148	91
HZY210	156	148	83
HZY1H	163	148	91
YY1787	161	148	91
PXYTZ	163	148	91
RY2117	155	148	91
CKYSM	163	136	63
JCY7021	159	136	103
SXY636	157	136	95
CY6203	163	136	95
DY4727	161	136	95
CKY637	163	136	95
TY068	157	136	63
FY498	157	128	71
TYXZ	156	128	71
LJY1706	156	128	71
N5YYX1H	155	128	71

**Table 2:** Rice yield and yield component of different cultivars under different cultivation systems.

Cultivar	Cultivation System	Grain Yield (t ha^−1^)	Change of Yield (%)	Panicle Number (×10^4^ ha^−1^)	Spikelets per Panicle	1000-Grain Weight (g)	Seed-Setting Rate (%)
YXY2115	SMR	8.10 ± 0.67 hi	0	387 ± 7 de	151 ± 13 efg	34.16 ± 0.19 a	92.19 ± 2.66 ab
	FR	6.78 ± 0.33 E	−16.26	563 ± 18 D	59 ± 2 H	32.76 ± 0.35 A	67.61 ± 3.09 FG
	MR	4.82 ± 0.13 *d*	−40.47	300 ± 9 *i*	89 ± 6 *cd*	31.43 ± 0.38 *b*	60.57 ± 1.40 *f*
CZY3607	SMR	9.48 ± 0.57 cde	0	512 ± 16 b	272 ± 19 a	22.79 ± 0.46 L	82.81 ± 2.20 def
	FR	5.97 ± 0.42 FG	−37.05	687 ± 22 B	80 ± 1 E	23.51 ± 0.47 J	78.04 ± 0.39 D
	MR	-	-	-	-	-	-
YXY2727	SMR	9.10 ± 0.35 defg	0	262 ± 6 j	185 ± 15 c	29.48 ± 0.17 d	74.26 ± 1.64 h
	FR	4.99 ± 0.08 IJ	−45.17	325 ± 13 JK	171 ± 7 A	29.32 ± 0.27 CD	78.52 ± 3.36 D
	MR	-	-	-	-	-	-
PXYWSSM	SMR	9.70 ± 0.71 abcd	0	425 ± 12 c	158 ± 11 def	22.84 ± 0.50 L	80.07 ± 2.94 efg
	FR	4.64 ± 0.19 JK	−52.19	462 ± 17 G	90 ± 3 CD	21.20 ± 0.30 M	50.79 ± 2.05 L
	MR	2.48 ± 0.12 *h*	−74.45	413 ± 22 *de*	67 ± 5 *i*	20.49 ± 0.31 *k*	44.06 ± 1.75 *h*
PXYMZ	SMR	9.33 ± 0.30 def	0	437 ± 16 c	158 ± 10 def	24.64 ± 0.20 j	76.73 ± 2.83 gh
	FR	2.23 ± 0.06 M	−76.14	300 ± 13 K	89 ± 2 CD	26.18 ± 0.44 G	41.16 ± 0.71 M
	MR	-	-	-	-	-	-
XLY16	SMR	9.33 ± 0.04 def	0	388 ± 13 de	155 ± 5 defg	23.59 ± 0.37 k	87.78 ± 4.31 abcd
	FR	6.36 ± 0.12 EF	−31.82	488 ± 14 FG	84 ± 4 DE	24.58 ± 0.19 I	68.28 ± 3.15 F
	MR	5.44 ± 0.29 *c*	−41.68	424 ± 9 *d*	93 ± 6 *bc*	23.69 ± 0.33 *g*	62.99 ± 0.51 *e*
GLYHZ	SMR	9.03 ± 0.69 defgh	0	373 ± 12 def	143 ± 7 fghij	21.47 ± 0.22 m	82.19 ± 2.29 def
	FR	5.70 ± 0.23 GH	−36.86	500 ± 24 EF	54 ± 3 H	20.96 ± 0.39 M	61.62 ± 2.23 HIJ
	MR	-	-	-	-	-	-
JLY8612	SMR	9.58 ± 0.61 bcde	0	337 ± 15 gh	166 ± 13 de	23.58 ± 0.15 k	76.50 ± 2.74 gh
	FR	5.96 ± 0.34 FG	−37.76	587 ± 15 D	71 ± 3 F	22.28 ± 0.19 L	55.80 ± 0.42 K
	MR	-	-	-	-	-	-
QY607	SMR	9.28 ± 0.66 def	0	325 ± 14 h	234 ± 13 b	23.73 ± 0.17 k	90.05 ± 2.87 abcd
	FR	4.42 ± 0.38 JK	−52.30	638 ± 12 C	80 ± 7 E	22.78 ± 0.3 KL	63.90 ± 0.52 HI
	MR	-	-	-	-	-	-
HZY210	SMR	8.68 ± 0.3 efghi	0	437 ± 19 c	124 ± 6 kl	23.75 ± 0.40 k	92.23 ± 4.30 ab
	FR	2.93 ± 0.17 L	−66.17	400 ± 15 H	54 ± 1 H	22.45 ± 0.21 L	40.45 ± 1.25 M
	MR	3.42 ± 0.27 *g*	−60.63	588 ± 15 *b*	48 ± 3 *k*	22.04 ± 0.29 *j*	54.88 ± 0.88 *g*
HZY1H	SMR	8.85 ± 0.28 defghi	0	387 ± 11 de	131 ± 10 hijkl	23.62 ± 0.19 k	79.21 ± 3.61 fgh
	FR	4.07 ± 0.08 K	−53.99	638 ± 17 C	42 ± 2 I	21.38 ± 0.25 M	70.59 ± 2.21 EF
	MR	-	-	-	-	-	-
YY1787	SMR	8.20 ± 0.50 ghi	0	338 ± 13 gh	139 ± 10 fghijk	28.73 ± 0.32 f	89.45 ± 3.72 abc
	FR	5.32 ± 0.24 HI	−35.18	362 ± 5 I	54 ± 4 H	29.47 ± 0.31 C	70.29 ± 2.68 EF
	MR	-	-	-	-	-	-
PXYTZ	SMR	9.48 ± 0.63 cde	0	375 ± 16 def	119 ± 3 l	28.91 ± 0.26 ef	85.32 ± 2.40 cde
	FR	2.47 ± 0.11 LM	−73.91	487 ± 19 FG	60 ± 4 GH	29.15 ± 0.27 CD	50.52 ± 1.53 L
	MR	-	-	-	-	-	-
RY2117	SMR	9.20 ± 0.48 def	0	325 ± 14 h	172 ± 7 cd	26.66 ± 0.37 h	82.67 ± 3.26 def
	FR	4.96 ± 0.07 IJ	−46.14	488 ± 18 FG	90 ± 7 CD	25.55 ± 0.10 H	64.47 ± 1.2 GH
	MR	-	-	-	-	-	-
CKYSM	SMR	10.58 ± 0.27 a	0	400 ± 9 d	218 ± 15 b	24.40 ± 0.34 j	86.41 ± 3.11 cd
	FR	7.83 ± 0.10 CD	−25.94	750 ± 27 A	93 ± 2 C	23.17 ± 0.17 JK	60.78 ± 1.76 IJ
	MR	3.59 ± 0.27 *g*	−66.06	325 ± 7 *h*	70 ± 5 *ghi*	28.39 ± 0.33 *c*	68.95 ± 2.91 *d*
JCY7021	SMR	8.80 ± 0.54 defghi	0	625 ± 23 a	129 ± 9 defg	29.27 ± 0.34 de	92.21 ± 2.53 ab
	FR	5.23 ± 0.31 HI	−40.60	500 ± 29 EF	60 ± 2 GH	28.95 ± 0.37 CDE	71.24 ± 2.99 EF
	MR	-	-	-	-	-	-
SXY636	SMR	8.43 ± 0.51 fghi	0	363 ± 13 efg	147 ± 9 fghi	27.95 ± 0.32 g	76.78 ± 3.27 gh
	FR	6.70 ± 0.39 E	−20.54	575 ± 9 D	53 ± 4 H	26.44 ± 0.33 G	82.31 ± 3.83 C
	MR	6.68 ± 0.08 *b*	−20.74	475 ± 27 *c*	79 ± 1 *ef*	27.60 ± 0.30 *d*	67.28 ± 2.50 *d*
CY6203	SMR	9.33 ± 0.35 def	0	388 ± 17 de	166 ± 9 de	27.11 ± 0.39 h	85.99 ± 4.04 cd
	FR	7.47 ± 0.32 D	−19.86	350 ± 18 IJ	96 ± 6 C	28.71 ± 0.22 DE	58.92 ± 0.52 JK
	MR	4.32 ± 0.26 *e*	−53.64	612 ± 13 *a*	69 ± 5 *hi*	25.80 ± 0.42 *f*	39.79 ± 1.11 *i*
DY4727	SMR	10.40 ± 0.44 ab	0	388 ± 25 de	138 ± 9 ghijk	32.30 ± 0.23 b	87.39 ± 4.55 abcd
	FR	6.70 ± 0.16 E	−35.61	400 ± 7 H	57 ± 3 H	31.83 ± 0.57 B	72.50 ± 1.05 E
	MR	5.43 ± 0.37 *c*	−47.83	350 ± 10 *g*	97 ± 6 *b*	32.62 ± 0.15 *a*	87.50 ± 1.67 *a*
CKY637	SMR	9.75 ± 0.23 abcd	0	300 ± 21 i	140 ± 10 fghijk	28.48 ± 0.31 f	87.69 ± 2.87 abcd
	FR	8.13 ± 0.53 C	−16.62	563 ± 19 D	73 ± 5 F	26.71 ± 0.36 G	70.54 ± 1.04 EF
	MR	3.54 ± 0.16 *g*	−63.71	387 ± 11 *f*	82 ± 1 *e*	26.37 ± 0.27 *e*	42.16 ± 1.59 *h*
TY068	SMR	9.18 ± 0.27 def	0	437 ± 11 c	146 ± 2 fghi	22.69 ± 0.27 L	86.61 ± 2.41 bcd
	FR	4.59 ± 0.19 JK	−49.99	737 ± 16 A	66 ± 5 FG	22.43 ± 0.50 L	85.93 ± 0.89 B
	MR	2.29 ± 0.14 *h*	−75.03	588 ± 11 *b*	84 ± 4 *de*	22.43 ± 0.46 *i*	42.42 ± 1.23 *h*
FY498	SMR	9.70 ± 0.62 abcd	0	300 ± 6 i	218 ± 5 b	26.73 ± 0.28 h	77.60 ± 2.96 fgh
	FR	9.65 ± 0.35 B	−0.49	425 ± 15 H	92 ± 3 C	29.33 ± 0.42 CD	69.75 ± 1.6 EF
	MR	3.62 ± 0.25 *g*	−62.68	325 ± 19 *h*	75 ± 5 *fgh*	26.62 ± 0.53 *e*	56.13 ± 1.08 *g*
TYXZ	SMR	9.43 ± 0.56 cde	0	275 ± 10 ij	149 ± 5 efgh	26.12 ± 0.24 i	92.35 ± 1.88 a
	FR	9.75 ± 0.57 B	+3.49	337 ± 9 IJ	129 ± 5 B	27.42 ± 0.36 F	89.86 ± 1.37 A
	MR	3.91 ± 0.12 *f*	−58.51	400 ± 11 *ef*	58 ± 2 *j*	25.77 ± 0.32 *f*	74.42 ± 2.07 *c*
LJY1706	SMR	10.35 ± 0.42 abc	0	325 ± 19 h	185 ± 7 c	23.60 ± 0.26 k	86.01 ± 2.39 cd
	FR	11.00 ± 0.79 A	+6.25	525 ± 22 E	71 ± 5 F	22.76 ± 0.17 KL	87.19 ± 1.27 AB
	MR	7.08 ± 0.37 *a*	−31.60	312 ± 4 *hi*	104 ± 7 *a*	25.96 ± 0.32 *f*	84.22 ± 3.89 *b*
N5YYX1H	SMR	8.03 ± 0.61 i	0	350 ± 19 fgh	127 ± 8 jkl	31.05 ± 0.26 c	88.72 ± 1.6 abc
	FR	6.23 ± 0.32 EFG	−22.41	463 ± 11 G	71 ± 5 F	28.37 ± 0.44 E	72.38 ± 1.78 E
	MR	3.39 ± 0.06 *g*	−57.77	616 ± 18 *a*	76 ± 3 *fg*	23.17 ± 0.20 *h*	31.38 ± 0.68 *j*

Notes: SMR, FR and MR represent single middle-season rice, first-season forage and second-season ratoon rice, and first middle-season rice and second-season ratoon rice, respectively. Lowercase letters indicate significant differences at *p* < 0.05 among cultivars when grown as single middle-season rice. Uppercase letters indicate significant differences at *p* < 0.05 among cultivars when grown as first-season forage and second-season ratoon rice. Italic letters indicate significant differences at *p* < 0.05 among varieties when grown as first middle-season rice and second-season ratoon rice. The data presented are the mean ± standard deviation, *n* = 3.

**Table 3:** Silage rice quality of different cultivars under different cultivation systems.

Cultivar	Starch (%)	Crude Protein (%)	Acid Detergent Fiber (%)	Neutral Detergent Fiber (%)	Dry Matter (%)	Ash (%)	Relative Feed Value
YXY2115	22.96 ± 1.14 b	9.26 ± 0.36 e	26.07 ± 1.60 e	33.97 ± 1.91 g	32.43 ± 1.33 a	15.91 ± 0.56 ab	188.36 ± 13.46 b
CKYSM	20.94 ± 1.07 c	12.31 ± 0.83 b	25.38 ± 0.81 e	54.48 ± 2.57 ab	19.60 ± 1.35 e	13.12 ± 0.46 d	118.21 ± 5.73 e
SXY636	15.20 ± 0.83 d	11.10 ± 0.72 c	33.20 ± 1.34 c	41.95 ± 1.89 d	21.13 ± 0.90 de	15.58 ± 0.92 ab	140.03 ± 8.46 d
XLY16	25.13 ± 1.42 a	10.69 ± 0.50 cd	19.97 ± 0.77 f	32.96 ± 0.72 f	28.97 ± 1.78 b	11.67 ± 0.59 e	207.05 ± 3.38 a
CY6203	3.33 ± 0.11 f	12.68 ± 0.91 b	35.36 ± 1.04 bc	52.70 ± 2.60 bc	23.31 ± 0.73 cd	16.72 ± 1.27 a	108.45 ± 4.95 e
DY4727	23.67 ± 0.87 ab	9.67 ± 0.59 de	27.13 ± 1.29 e	32.03 ± 1.41 f	24.92 ± 1.65 c	15.39 ± 0.61 ab	196.96 ± 6.20 ab
CKY637	2.96 ± 0.08 f	8.68 ± 0.42 e	52.82 ± 2.38 a	57.67 ± 3.14 a	21.01 ± 1.51 de	13.62 ± 0.31 cd	77.08 ± 1.47 f
FY498	23.16 ± 1.36 b	9.26 ± 0.35 e	26.50 ± 0.68 e	53.23 ± 1.30 bc	22.82 ± 1.08 cd	15.08 ± 0.93 b	119.34 ± 3.75 e
TYXZ	3.31 ± 0.10 f	15.72 ± 0.85 a	33.78 ± 1.19 c	51.80 ± 2.04 bc	20.17 ± 1.41 e	15.91 ± 0.57 ab	112.53 ± 5.66 e
LJY1706	3.93 ± 0.17 f	13.33 ± 0.34 b	36.53 ± 1.11 b	49.95 ± 1.46 c	19.49 ± 1.03 e	16.82 ± 0.95 a	112.60 ± 2.91 e
N5YYX1H	12.83 ± 0.57 e	12.76 ± 0.64 b	29.80 ± 1.91 d	38.03 ± 1.42 e	24.46 ± 0.32 c	14.71 ± 0.69 bc	160.86 ± 8.72 c

Notes: SMR, FR and MR represent single middle-season rice, first-season forage and second-season ratoon rice, and first middle-season rice and second-season ratoon rice, respectively. Lowercase letters indicate significant differences at *p* < 0.05 among cultivars when grown as single middle-season rice. The data presented are the mean ± standard deviation, *n* = 3.

**Table 4:** The scores and rankings of different cultivars.

Cultivar	Score	Ranking
CKY637	0.614	1
CY6203	0.609	2
CKYSM	0.476	3
LJY1706	0.371	4
FY498	0.359	5
DY4727	0.249	6
TYXZ	0.129	7
SXY636	−0.026	8
XLY16	−0.715	9
N5YYX1H	−0.787	10
YXY2115	−1.279	11

## Data Availability

The data presented in this study are available on request from the corresponding author. The data are not publicly available due to privacy and ethical restrictions.

## Supplementary Materials

The following supporting information can be downloaded at: https://www.mdpi.com/article/10.3390/plants15172725/s1, Table S1: Soil properties of the topsoil layer (0–0.20 m) at the experimental sites; Table S2: Detailed information of different rice cultivars; Table S3: Stubble number of different cultivars under forage-grain dual-purpose ratoon rice system; Table S4: Ratooning ability of different cultivars under forage-grain dual-purpose ratoon rice system; Table S5: Stubble survival rate of different cultivars under forage-grain dual-purpose ratoon rice system; Table S6: 5 indicators used for principle component analysis; Table S7: The scores and rankings of different cultivars; Figure S1: Temperature and rainfall during the experimental periods.
