# Supplementary material for: Evaluation and Selection of Rice Cultivars for Dual-Purpose Grain and Forage Production System
Source: Plants (Basel). 2026 Sep 6;15(17):2725. doi: 10.3390/plants15172725 (PMC13567482; doi:10.3390/plants15172725)
Supplement: Supplementary file 1 [file plants-15-02725-s001.zip › plants-4501159-supplementary.pdf]

Table S1 Soil properties of the top soil layer (0-0.20 m) at the experimental sites

Year	Organic matter ($g\ kg^{-1}$)	Total N ($g\ kg^{-1}$)	Total P ($g\ kg^{-1}$)	Total K ($g\ kg^{-1}$)	Available N ($mg\ kg^{-1}$)	Available P ($mg\ kg^{-1}$)	Available K ($mg\ kg^{-1}$)	pH
2024	30.31	1.58	0.36	15.32	114.15	10.08	139.31	6.37

N, P, and K represent nitrogen, phosphorus and potassium, respectively.

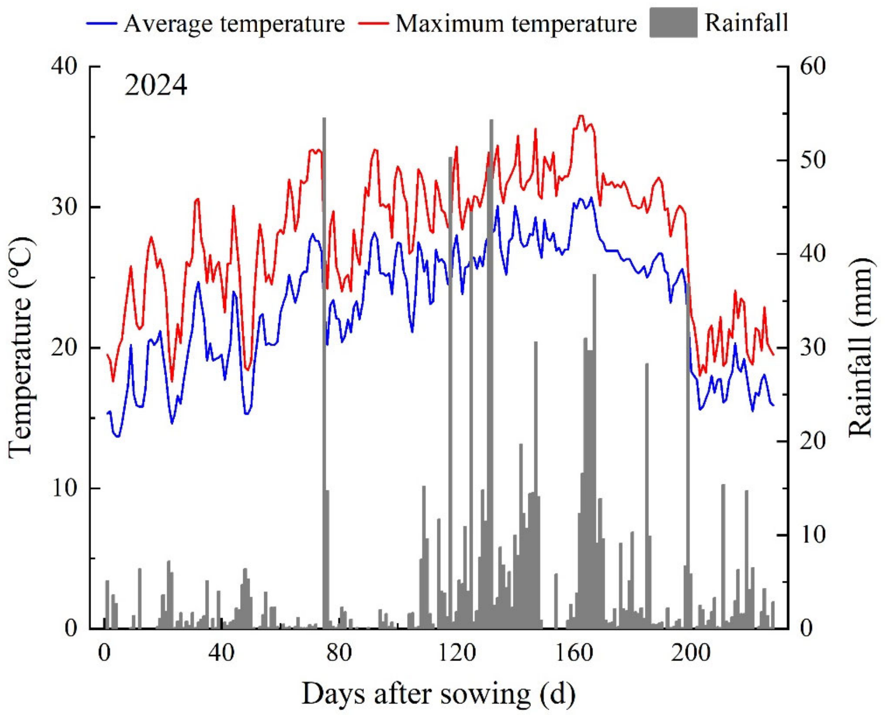

Figure S1 Temperature and rainfall during the experimental periods

Table S2 Detailed information of different rice cultivars

Number	Cultivar	Type	Approval year	Growth duration (d)	Yield (t ha^{-1})	Plant height (cm)	Approximate cumulative promotion area (ha)
1	Yixiangyou 2115<br>YXY2115	Indica three-line hybrid rice	2021	146.0	8.30	124.0	2,000,000
2	Chuanzhongyou 3607<br>CZY3607	Indica three-line hybrid rice	2020	152.7	10.02	110.3	23,333
3	Yuxiangyou 2727<br>YXY2727	Indica three-line hybrid rice	2021	151.7	9.61	120.3	10,000
4	Pinxiangyou Wushan Simiao<br>PXYWSSM	Indica three-line hybrid rice	2022	131.3	9.72	118.6	5,333
5	Pinxiangyou Meizhen<br>PXYMZ	Indica three-line hybrid rice	2021	156.8	8.39	111.3	26,667
6	Xiangliangyou 16<br>XLY16	Indica two-line hybrid rice	2021	145.5	8.57	114.1	10,000
7	Guanliangyou Huazhan<br>GLYHZ	Indica two-line hybrid rice	2020	134.2	9.66	118.2	5,333
8	Jingliangyou 8612<br>JLY8612	Indica two-line hybrid rice	2021	147.4	8.80	117.1	13,333
9	Quanyou 607<br>QY607	Indica three-line hybrid rice	2022	154.1	9.84	118.1	150,000
10	Huazheyou 210<br>HZY210	Indica three-line hybrid rice	2023	121.1	9.30	131.6	6,667
11	Huazheyou 1 Hao<br>HZY1H	Indica three-line hybrid rice	2020	134.5	7.94	104.6	5,333
12	Yiyou 1787<br>YY1787	Indica three-line hybrid rice	2023	131.1	8.05	124.6	10,000
13	Pinxiangyou Tongzhen<br>PXYTZ	Indica three-line hybrid rice	2023	132.9	9.37	125.3	100,000
14	Ruiyou 2117<br>RY2117	Indica three-line hybrid rice	2020	150.4	9.62	118.5	5,333
15	Chuankangyou Simiao<br>CKYSM	Indica three-line hybrid rice	2019	149.4	9.56	114.2	66,667
16	Jinchengyou7021<br>JCY7021	Indica three-line hybrid rice	2021	151.5	8.75	117.8	5,333
17	Shuxiangyou636<br>SXY636	Indica three-line hybrid rice	2019	151.3	9.21	125.4	10,000
18	Chuanyou 6203<br>CY6203	Indica three-line hybrid rice	2014	131.8	9.39	122.9	150,000
19	Deyou 4727<br>DY4727	Indica three-line hybrid rice	2014	149.4	8.45	116.4	10,000
20	Chuankangyou 637<br>CKY637	Indica three-line hybrid rice	2020	150.0	10.09	116.5	10,000
21	Taiyou 068<br>TY068	Indica three-line hybrid rice	2020	152.0	9.72	111.7	5,333
22	F You 498<br>FY498	Indica three-line hybrid rice	2011	155.2	8.74	111.9	333,333
23	Taoyou Xiangzhan<br>TYXZ	Indica three-line hybrid rice	2021	124.9	8.76	114.0	233,333
24	Longjingyou 1706<br>LJY1706	Indica three-line hybrid rice	2021	125.9	8.80	116.8	10,000
25	Nei 5 You Yuxiang 1 Hao<br>N5YYX1H	Indica three-line hybrid rice	2012	112.2	7.10	113.3	10,000

Note: The values are approximate cumulative promotion areas estimated from publicly available information and are not intended to represent annual planting areas.

Table S3 Stubble number of different cultivars under forage-grain dual-purpose ratoon rice system

Cultivar	Stubble height 5 cm	Stubble height 15 cm	Stubble height 25 cm
YXY2115	366.67±29.40fgh	577.78±44.44c	311.11±22.22k
CZY3607	377.78±11.11efgh	507.41±16.97def	340.74±27.96kl
YXY2727	422.22±33.33de	455.56±40.06efg	444.44±22.22fghi
PXYWSSM	577.78±29.40a	577.78±55.56c	544.44±40.06b
PXYMZ	544.44±29.40a	477.78±22.22efgh	488.89±11.11cdef
XLY16	577.78±29.40a	433.33±33.33ghi	422.22±22.22ghij
GLYHZ	588.89±33.33a	655.56±29.40b	488.89±44.44cdef
JLY8612	477.78±22.22bc	522.22±44.44cd	533.33±19.25bc
QY607	333.33±11.11hi	388.89±33.33hij	466.67±11.11defg
HZY210	422.22±33.33de	455.56±33.33efg	500.00±29.40bcde
HZY1H	544.44±29.40a	433.33±22.22ghi	411.11±22.22hij
YY1787	333.33±29.40hi	355.56±19.25jk	377.78±29.40j
PXYTZ	311.11±19.25i	400.00±19.25ghij	377.78±22.22j
RY2117	444.44±22.22cd	433.33±33.33ghi	377.78±22.22j
CKYSM	577.78±19.25a	433.33±29.40ghi	488.89±40.06cdef
JCY7021	366.67±22.22fgh	511.11±19.25de	488.89±29.40cdef
SXY636	411.11±22.22def	311.11±11.11k	311.11±22.22k
CY6203	500.00±22.22b	500.00±40.06def	400.00±33.33ij
DY4727	500.00±29.40b	366.67±22.22jk	377.78±22.22j
CKY637	355.56±33.33ghi	377.78±29.40ij	400.00±19.25ij
TY068	577.78±19.25a	777.78±58.79a	855.56±40.06a
FY498	388.89±19.25efg	377.78±33.33ij	277.78±11.11k
TYXZ	544.44±29.40a	555.56±11.11cd	522.22±33.33bc
LJY1706	555.56±38.49a	444.44±22.22fgh	511.11±22.22bcd
N5YYX1H	388.89±11.11efg	377.78±22.22ij	455.56±11.11efgh

Note: Different lowercase letters in the same column indicate significant difference among treatments at $p < 0.05$. The data presented are the mean ± standard deviation, $n = 3$.

Table S4 Ratooning ability of different cultivars under forage-grain dual-purpose ratoon rice system

Cultivar	Stubble height 5 cm	Stubble height 15 cm	Stubble height 25 cm
YXY2115	1.56±0.20a	0.77±0.07i	1.18±0.08fgh
CZY3607	1.46±0.09abc	0.92±0.01hi	1.20±0.03fgh
YXY2727	1.48±0.20ab	1.05±0.05gh	1.10±0.11fgh
PXYWSSM	1.33±0.01bcdef	1.07±0.13gh	0.98±0.04hij
PXYMZ	1.25±0.12cdefg	1.06±0.04gh	0.97±0.07hij
XLY16	1.16±0.11efgh	1.14±0.17fgh	0.82±0.01j
GLYHZ	1.33±0.15bcdef	1.53±0.09abc	2.01±0.14a
JLY8612	1.40±0.11abcd	1.71±0.15a	1.77±0.17bc
QY607	1.40±0.05abcd	1.15±0.11efgh	0.88±0.06ij
HZY210	1.00±0.07hi	1.12±0.03fgh	0.82±0.04j
HZY1H	1.49±0.15ab	1.60±0.20ab	1.92±0.16ab
YY1787	1.31±0.11bcdef	0.97±0.08hi	1.24±0.15fg
PXYTZ	1.32±0.11bcdef	1.00±0.05h	1.12±0.06fgh
RY2117	0.90±0.01i	1.24±0.17defg	1.33±0.10ef
CKYSM	1.19±0.10defgh	1.57±0.04abc	1.63±0.23cd
JCY7021	1.25±0.12cdefg	1.11±0.08fgh	0.77±0.02j
SXY636	1.14±0.05fgh	1.50±0.11abc	1.15±0.18fgh
CY6203	1.38±0.09abcd	1.61±0.19ab	1.65±0.20cd
DY4727	1.13±0.02fgh	1.64±0.06ab	1.68±0.16cd
CKY637	1.14±0.18fgh	1.68±0.17ab	1.58±0.03cd
TY068	1.40±0.06abcd	1.45±0.22bcd	1.26±0.10fg
FY498	1.34±0.11bcdef	1.33±0.19cdef	1.52±0.16de
TYXZ	1.35±0.07abcde	1.36±0.10cdef	1.58±0.19cd
LJY1706	1.43±0.09abc	1.45±0.07bcd	1.50±0.10de
N5YYX1H	1.09±0.10gh	1.03±0.09gh	1.05±0.09ghi

Note: Different lowercase letters in the same column indicate significant difference among treatments at $p < 0.05$. The data presented are the mean ± standard deviation, $n = 3$.

Table S5 Stubble survival rate of different cultivars under forage-grain dual-purpose ratoon rice system

Cultivar	Stubble height 5 cm	Stubble height 15 cm	Stubble height 25 cm
YXY2115	100.00±0.00a	100.00±0.00a	100.00±0.00a
CZY3607	93.16±1.50ef	98.57±1.24ab	95.65±1.84c
YXY2727	92.07±0.63e	97.55±0.22b	94.99±0.25c
PXYWSSM	92.29±0.40e	94.19±0.56c	85.66±1.03f
PXYMZ	94.59±0.92d	92.24±1.37d	92.40±1.48e
XLY16	94.22±0.30d	92.28±0.60d	92.09±0.42d
GLYHZ	96.22±0.21c	100.00±0.00a	100.00±0.00a
JLY8612	100.00±0.00a	100.00±0.00a	97.91±0.08ab
QY607	100.00±0.00a	94.26±0.49c	80.95±0.45g
HZY210	73.64±2.48i	92.66±0.54d	79.95±1.21g
HZY1H	100.00±0.00a	100.00±0.00a	100.00±0.00a
YY1787	100.00±0.00a	84.35±0.82f	97.05±0.22b
PXYTZ	96.42±0.21c	77.66±3.83g	97.05±0.17b
RY2117	70.03±1.00j	92.28±0.60d	70.47±3.97i
CKYSM	92.30±0.25e	100.00±0.00a	100.00±0.00a
JCY7021	87.85±0.74f	89.12±0.40e	74.88±3.44h
SXY636	86.46±0.73g	100.00±0.00a	100.00±0.00a
CY6203	100.00±0.00a	100.00±0.00a	100.00±0.00a
DY4727	84.48±1.65h	100.00±0.00a	97.05±0.17b
CKY637	87.43±1.18fg	100.00±0.00a	100.00±0.00a
TY068	100.00±0.00a	100.00±0.00a	93.50±0.30cd
FY498	100.00±0.00a	100.00±0.00a	100.00±0.00a
TYXZ	93.87±0.34d	100.00±0.00a	100.00±0.00a
LJY1706	97.99±0.14b	100.00±0.00a	100.00±0.00a
N5YYX1H	88.57±0.33f	85.26±0.87f	90.24±0.24e

Note: Different lowercase letters in the same column indicate significant difference among treatments at $p < 0.05$. The data presented are the mean ± standard deviation, $n = 3$.

Table S6 5 indicators used for principle component analysis

Cultivar	Original data					Standardized processed data				
	Silage rice yield	Ratooning ability	Grain Cd concentration	Ratoon rice yield	Relative feed value	Silage rice yield	Ratooning ability	Grain Cd concentration	Ratoon rice yield	Relative feed value
YXY2115	6.7836	0.7727	0.1105	12.1090	188.3586	0.1167	0.0000	0.0000	0.3755	0.8562
XLY16	6.3585	1.1361	0.0762	13.5982	207.0516	0.0276	0.3992	0.4655	0.5265	1.0000
CKYSM	7.8321	1.5655	0.0457	11.2689	118.2129	0.3365	0.8708	0.8798	0.2903	0.3165
SXY636	6.6953	1.5022	0.0873	13.1261	140.0348	0.0982	0.8012	0.3154	0.4787	0.4844
CY6203	7.4740	1.6101	0.0368	12.0186	108.4509	0.2614	0.9198	1.0000	0.3664	0.2414
DY4727	6.6968	1.6385	0.0548	18.2673	196.9580	0.0985	0.9509	0.7559	1.0000	0.9223
CKY637	8.1303	1.6832	0.0686	8.8492	77.0818	0.3990	1.0000	0.5683	0.0450	0.0000
FY498	9.6538	1.3347	0.0430	9.3443	119.3366	0.7183	0.6173	0.9160	0.0952	0.3251
TYXZ	9.7547	1.3607	0.0872	8.4058	112.5306	0.7395	0.6459	0.3164	0.0000	0.2727
LJY1706	10.9975	1.4503	0.0967	10.0374	112.6024	1.0000	0.7442	0.1872	0.1654	0.2733
N5YYX1H	6.2270	1.0330	0.0728	8.5534	160.8629	0.0000	0.2858	0.5121	0.0150	0.6446

Table S7 The scores and rankings of different cultivars

Cultivar	Ranking	Score	Principle component 1 (F_1)	Principle component 2 (F_2)	Principle component 3 (F_3)
CKY637	1	0.614	1.168	0.113	-0.228
CY6203	2	0.609	0.574	1.166	-0.754
CKYSM	3	0.476	0.531	0.779	-0.560
LJY1706	4	0.371	0.932	-0.984	1.795
FY498	5	0.359	0.874	0.000	-0.720
DY4727	6	0.249	-1.068	1.825	1.227
TYXZ	7	0.129	0.808	-0.995	0.468
SXY636	8	-0.026	-0.373	0.242	0.629
XLY16	9	-0.715	-1.374	0.045	-0.148
N5YYX1H	10	-0.787	-0.576	-0.728	-1.783
YXY2115	11	-1.279	-1.496	-1.463	0.075
